# The Chemistry of Short-Lived α-Fluorocarbocations

**DOI:** 10.1021/acs.joc.0c02731

**Published:** 2021-02-22

**Authors:** Shlomo Rozen, Inna Vints, Ana Lerner, Oded Hod, Edward N. Brothers, Salvador Moncho

**Affiliations:** † School of Chemistry, Tel Aviv University, Tel Aviv 6997801, Israel; ‡ Department of Physical Chemistry, School of Chemistry, The Raymond and Beverly Sackler Faculty of Exact Sciences and The Sackler Center for Computational Molecular and Materials Science, Tel Aviv University, Tel Aviv 6997801, Israel; § Science Program, Texas A&M University at Qatar, Education City, Doha 23874, Qatar; ∥ Chemistry Department, Ben-Gurion University of the Negev, Beer-Sheva 84105, Israel

## Abstract

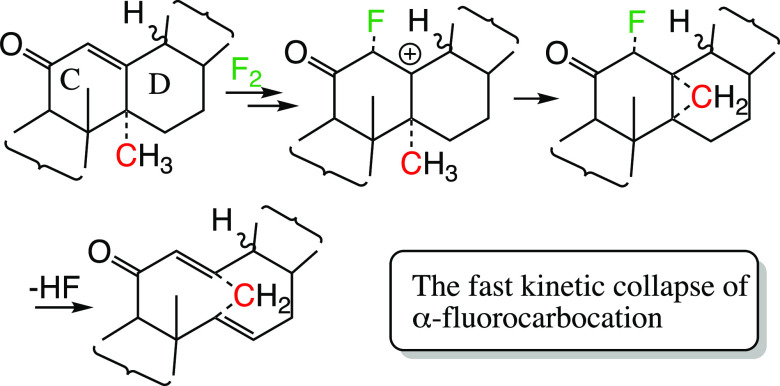

The present study of the chemistry of short-lived α-fluorocarbocations reveals that even inactive methyl carbons can serve as nucleophiles, attacking a cationic center. This, in turn, facilitates the synthesis of a cyclopropane ring in certain triterpene backbones. We report the synthesis of compounds similar to **2**, containing a bridgehead cyclopropane, and compounds of type **3** with an 11 membered bicyclic ring consisting of two bridgehead double bonds (anti-Bredt) within a triterpene skeleton. The synthesis involves three unconventional chemical processes: (a) a methyl group serving as a nucleophile; (b) the unexpected and unprecedented synthesis of a strained system in the absence of an external neighboring trigger; and (c) the formation of an 11-membered bicyclic diene ring within a triterpenoid skeleton. An α-fluorocarbocation mechanism is proposed and supported by density functional theory calculations.

## Introduction

Following carbon, oxygen, hydrogen, and nitrogen, fluorine stands out due to its vast impact on medicinal chemistry, agriculture, material science, modern anesthetics, refrigerants, and more.^1^ As such, it is desirable to identify distinctive fluorine-based intermediate structures that may serve as platforms for the synthesis of novel compounds and unveil their unique chemistry.

α-Fluorocarbocations constitute an important family of such fluorine-bearing intermediates. Nevertheless, only a limited number of reactions that involve this halo-carbocation are known.^2^ Because fluorine is the most electronegative atom, α-fluorocarbocation intermediates are expected to be considerably more reactive than the corresponding bare carbocations of different types^3^ and hence exhibit significantly shorter lifetimes. This, in turn, opens the door for novel α-fluorocarbocation-based chemistry that requires broad understanding of their nature. In the present study, we demonstrate and analyze examples of unusual reactions that take place when this intriguing species serves as an intermediate.

**Figure 1:**
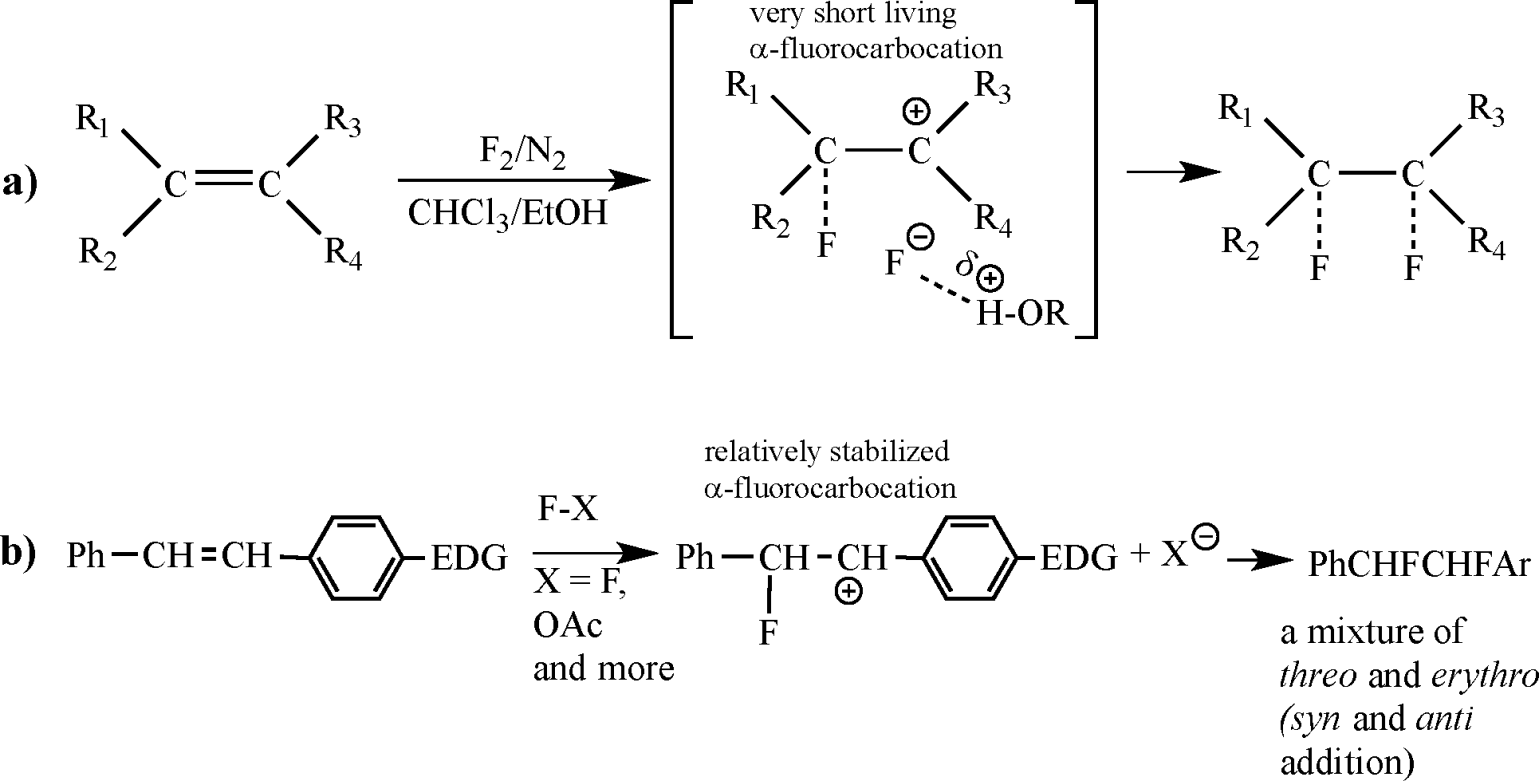
Examples of chemical reactions where an intermediate α-fluorocarbocation is formed via the attack of a double bond by an electrophilic fluorine. (a) A standard parallel attack of F_2_ on a double bond resulting in a short-lived α-fluorocarbocation that forms a *syn*-addition product. (b) By stabilizing the α-fluorocarbocation, both *syn*- and *anti*-addition products were obtained.^2,5^

Previously, some of us have shown that under suitable conditions, nitrogen diluted fluorine can be a suitable starting point for many new and unprecedented reactions,^4^ including *syn* addition across both isolated double bonds and enones.^5^ Unlike the heavier halogens, elemental fluorine as well as other reagents possessing a strong electrophilic fluorine atom such as acetyl- and trifluoroacetyl hypofluorites add across those π centers in a *syn* mode through a fast reaction.^2,3^ The underlying mechanism involved the initial formation of a very short-lived ion pair cage constituting of the α-fluorocarbocation and its counterion (Figure 1a) that instantly collapsed into the *syn* product. A four-center reaction mechanism was ruled out because when stabilizing the α-fluorocarbocation (as in 4-methoxystylbene), a mixture of stereoisomers resulting from both *syn-* and *anti*-additions was obtained (Figure 1b).^2^ Obviously the latter cannot be a product of a four-center process. Because these reactions have been performed at low temperatures and in the presence of polar solvents and/or radical inhibitors, a radical mechanism was also ruled out, suggesting an ionic mode of addition.^6^

## Results and Discussion

**Figure 2:**
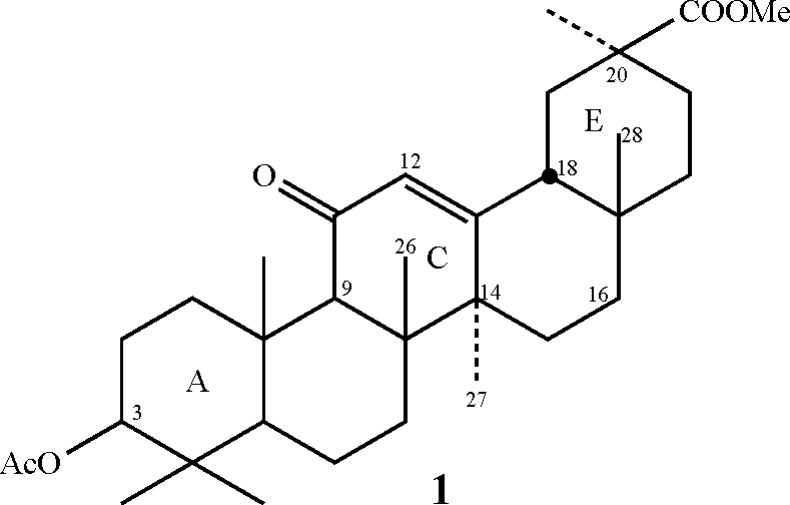
Molecular structure of methyl 3-acetoxy-18β-glycyrrhetate

Unlike the above findings, there are cases where the F_2_ cannot approach the targeted double bond in a parallel configuration. A distinctive example of such a situation can be found in several important triterpene molecules, such as methyl 3-acetoxy-18β-glycyrrhetate (**1**),^7^ a member of the β-amirin family (see Figure 2). Interest in this specific compound arose mainly due to its biological activities, especially against various ulcers.^8^ This led to extensive explorative modifications of its skeleton^9^ none, however, around the remarkably stable enone in ring C. Notably, even when the highly reactive [BrF] types of reagents are present, the enone in **1** remains unaffected^10^ because the bulky bromine atom cannot readily access the C12 position. This supports the argument that when a fluorine molecule attacks this bond, its ability to approach it in the preferred parallel orientation is inhibited by steric effects associated with the nearby methyl groups (see further discussion of this issue in SI Section 2 and below).

**Figure 3:**
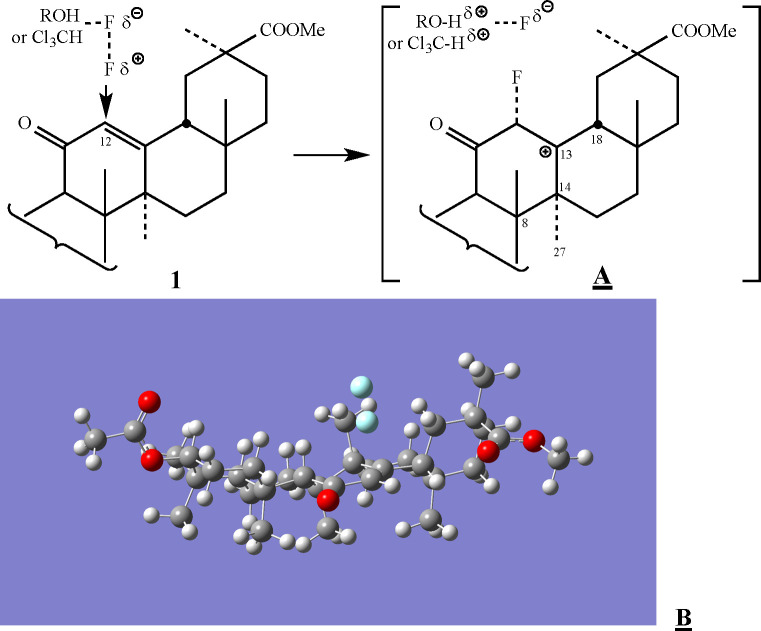
Formation of the α-fluorocarbocation cage of **1**. (A) Schematic representation of the vicinity of the reaction center. (B) The outcome of the calculated path to the transition state as obtained by the DFT calculations.

In the present study, to avoid radical reactions (and thus mixtures of different compounds) and to encourage an ionic mechanism that promotes more distinctive products, we dissolved **1** in a polar solvent mixture (CHCl_3_/CFCl_3_/EtOH 1/1/0.2) and reacted it with F_2_ (10% in N_2_) at −75 °C. Under these conditions, we expected the positive pole of the polarized small fluorine molecule to easily attack the enone of the C ring in an electrophilic mode.^11^ However, as mentioned above, the fluorine cannot approach the enone in a parallel orientation, so instead, it attacks carbon 12 in a nearly perpendicular mode (see Figure 3). This hypothesis is further supported by density functional theory (DFT) calculations (see below). Such an action by the fluorine results in a cage of ions (structure **A** in Figure 3) where the fluoride anion, which is stabilized by the acidic hydrogens of the solvent, is relatively far from the positively charged carbon of the α-fluorocarbocation. This, in turn, implies that unlike the case of isolated double bonds or common enones, the resulting very short-lived cage containing the α-fluorocarbocation **A** cannot easily form *syn*-difluoro derivatives and must find an alternative route to collapse.

**Figure 4:**
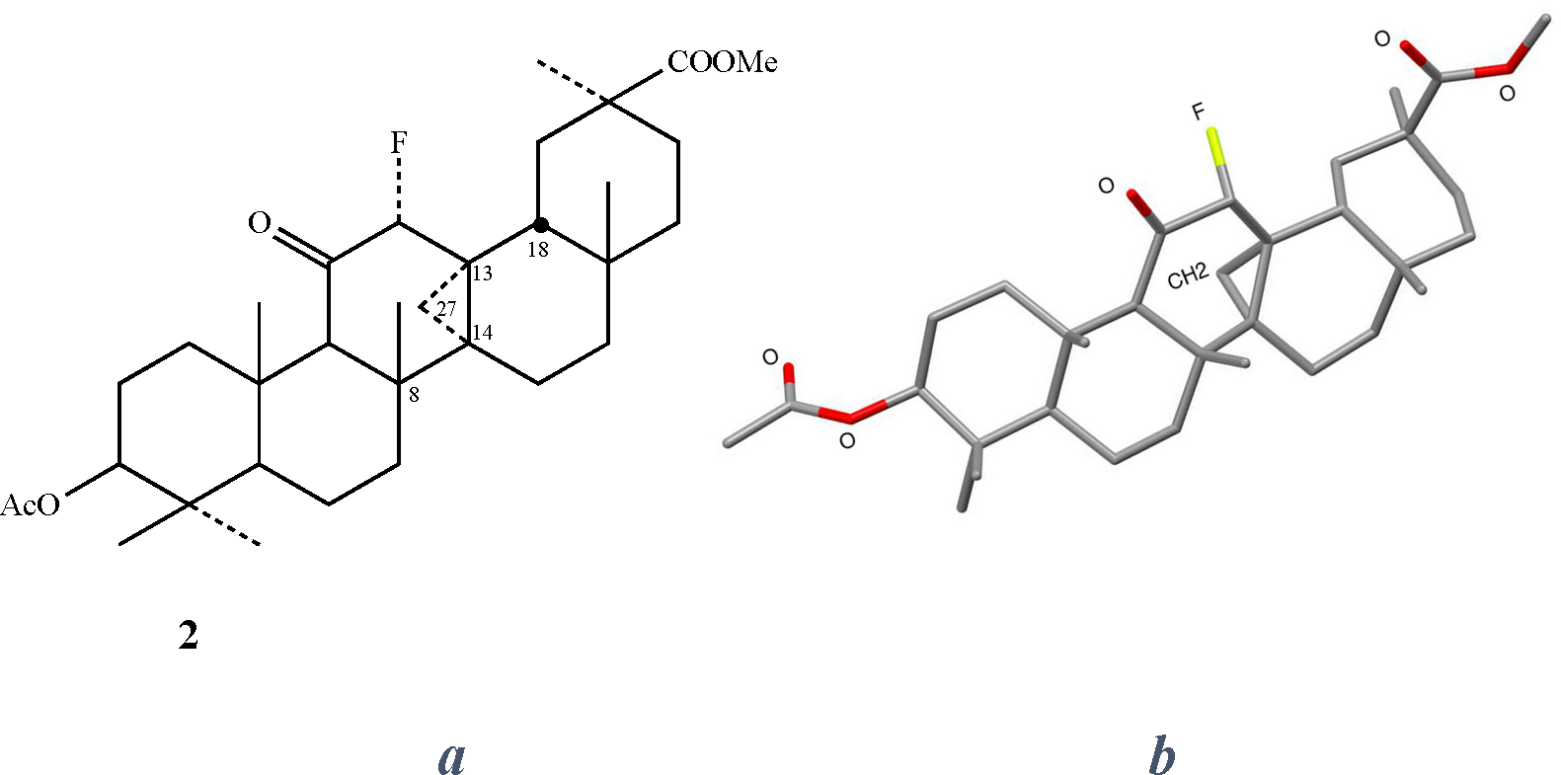
(a) Schematic and (b) X-ray structure of the cyclopropane derivative **2**.

Intuitively, one may envision two plausible pathways for the collapse mechanism of the short-lived fluorocarbocation in cage **A** involving the ejection of either the hydrogen at the C12 or at the C18 position to form the corresponding enone. Surprisingly, neither of these pathways is undertaken. Instead, an unprecedented nucleophilic attack on the α-fluorocarbocation at C13 by the carbon of the angular 27-methyl group takes place. This attack is accompanied by the ejection of a proton from that methyl and the formation of a strained cyclopropane derivative (**2**) with greater than 90% yield (Figure 4).

To substantiate this unexpected finding, we measured the ^1^H NMR spectrum of **2** (see SI Section 1), which demonstrates the disappearance of the 27-methyl group signature, usually found at 1.22 ppm, and the appearance of a single cyclopropane hydrogen at 0.47 ppm. The second cyclopropyl hydrogen could not be identified as it is somewhat deshielded by the α-fluoro-carbonyl, thus appearing in a region where many other methylene hydrogens resonate. This is supported by our calculated NMR signals yielding a 1.31 ppm peak for the 27-methyl group of **1** and 0.37 and 1.38 ppm peaks for the cyclopropane hydrogens of **2** (see Figure S1 in SI Section 2 for the calculated NMR spectrum). Further validation of our findings, is given by the X-ray diffraction measurement of compound **2** (Figure 4b) which unequivocally demonstrates the cyclopropane structure (for the cif file, see SI Section 3).

**Figure 5:**
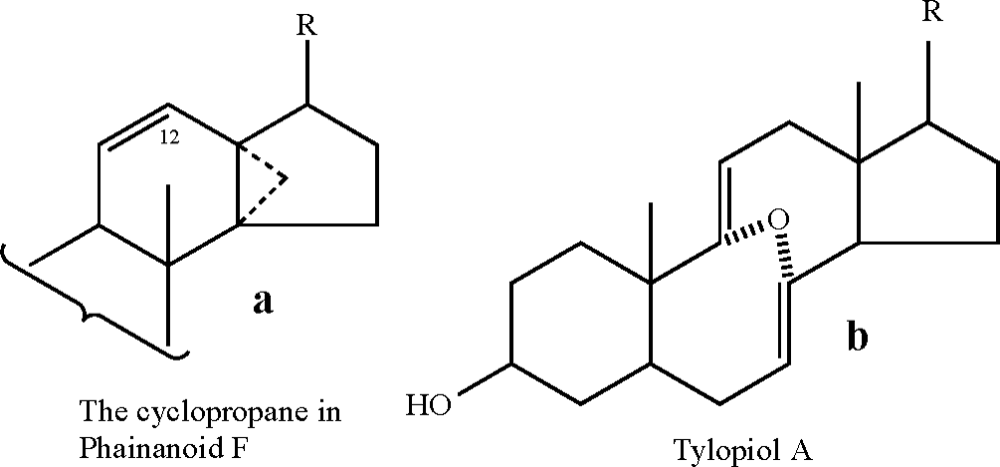
The only naturally occurring (to the best of our knowledge) structures that resemble those synthesized in the present study. (a) A cyclopropane ring within the naturally occurring phainanoid F. (b) An oxo-diene bridged 11-membered bicyclic ring in the naturally occurring Tylopiol A.

This unique product strongly indicates the stereoelectronic factors facilitating the involvement of the 27-methyl group, which kinetically favors the cyclopropane structure formation over the thermodynamically preferred enone products. To the best of our knowledge, the only other triterpenoid possessing a cyclopropane ring between rings C and D is the recently isolated phainanoid F (see Figure 5a for a partial structure), which was found to be a stout immunosuppressant.^12^ Therefore, exploiting this mechanism not only unveils unique chemistry but also opens a new route to biologically relevant structural motifs.

To elucidate the underlying mechanism for the formation of the cyclopropane species, we performed a set of DFT calculations characterizing the reaction pathway at the ωB97XD^13^/6-31+g(d,p) level of theory using the Gaussian Development Version^14^ (see SI Section 2 for further details regarding the calculations). In theory, the approach of the F_2_ toward the triterpene’s enone moiety could proceed from either side of the triterpene’s plane with a slight preference of the α-side, and since the experimental findings indicate that the attack is indeed from that direction, we proceeded with the DFT calculations accordingly (the data regarding the β-side reaction is available in Figure S3 of SI Section 2).

To explore the entire reaction path between the reactants and the cyclopropanated species (**2**), we performed intrinsic reaction coordinate (IRC) analysis.^15^ We find that after 16 steps of the IRC procedure, the perpendicular approach of the fluorine results in C12–F bond formation with bond length of 1.387 Å, which is the most stable isolated α-fluorocarbocation geometry. The accompanied fluoride anion is practically free (2.180 Å from the other fluorine) (see SI Section 2, Figures S4 and S5).

**Figure 6:**
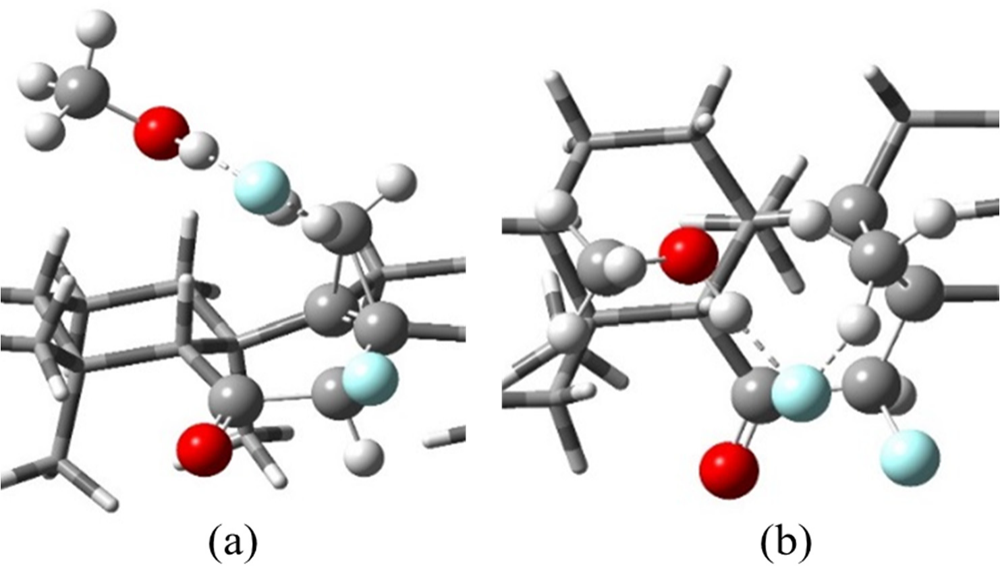
Side (a) and top (b) views of the DFT optimized geometry of the α-fluorocarbocation/fluoride ion pair optimized with an implicit chloroform solvent model in the presence of an explicit methanol molecule. For clarity, only the relevant region is depicted.

As was recently demonstrated by Singleton,^16^ however, calculations with explicit solvent molecules are important for concluding the exact effects on ion pair stability. To evaluate the role of the chloroform/ethanol solvent on the reaction mechanism, we performed calculations with a minimalistic explicit solvent model. To this end, the α-fluorocarbocation/fluoride ion pair was placed in an implicit chloroform solvent environment and optimized in the presence of a methanol molecule (see Figure 6). An intermediate structure prior to the C27 methyl hydrogen abstraction is found. In this intermediate, the distance between the fluoride anion and the C27 methyl’s hydrogen is 1.680 Å, while the corresponding C–H bond is elongated to 1.129 Å. The formation of such a structure points toward a two-step pathway. The competition with the alcohol molecule results in weakening of the methyl C27–H bond, which by itself is significantly tilted toward the α-fluorocarbocation, but with no full cleavage. The second inevitable step would be the final construction of the cyclopropane. Therefore, even in the presence of an explicit solvent that reduces the tendency of the leaving fluoride anion to accept one of the C27 methyl hydrogens, the short live α-fluorocarbocation tends to undergo a fast (though not completely barrierless) cyclopropanation reaction.

Having established the reliability of the DFT calculations for the studied system, we harnessed the same computational protocol to perform a reference calculation, where the F_2_ is replaced by HF forming a similar, but fluorine-free carbocation. This allows us to evaluate the importance of the fluorine atom within the molecular backbone for the intramolecular cyclopropanation reaction. Upon attack on the double bond, the proton of the HF molecule is inserted in position C12, forming a plain carbocation while the fluoride anion leaves. The corresponding geometry and charge distribution are given in SI Section 2, Table S2, and Figure S6. As in the case of the F_2_ attack, the carbocation/fluoride anion pair could potentially result in the fluoride abstracting a hydrogen atom from either the C27 methyl group or the C14 or C18 positions. In particular, the cyclopropanated species of the HF attack is 7.3 kcal/mol less stable than the reactants (to be compared with the corresponding F_2_ structures, which is about ∼100 kcal/mol more stable than the reactants). This indicates that the thermodynamically favorable option in the case of HF attack is to decompose back to the reactants. Furthermore, the barrier to cross during this endoergic reaction is extremely high (41.2 kcal/mol compared to 2.7 kcal/mol for the F_2_ attack case), marking it also as kinetically unfavorable. Therefore, we conclude that during the reaction with F_2_, the fluorine insertion to the double bond leading to the short-lived α-fluorocarbocation is essential for the rapid cyclopropane moiety formation.

**Figure 7:**
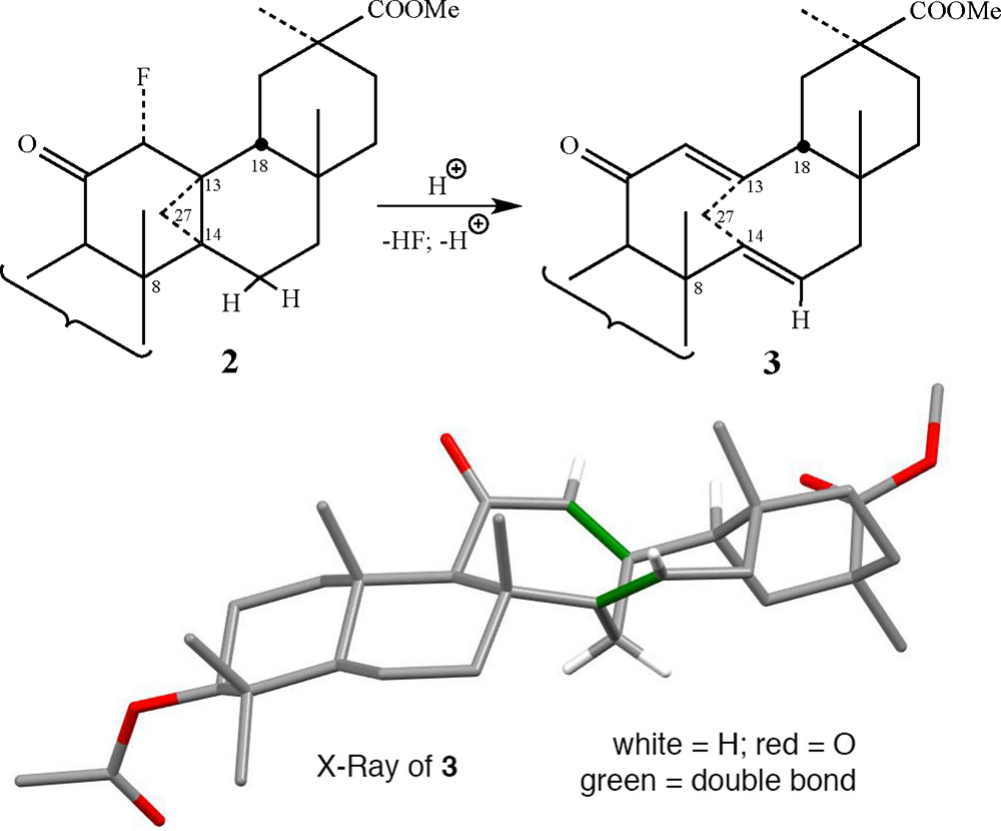
(top) The reaction of **2** converted to **3** and (bottom) the X-ray structure of **3**.

Once formed, the cyclopropanated structure **2** is obviously a metastable structure. We found that the presence of solvents possessing even weak acidic hydrogens, such as chloroform, is sufficient to promote the opening of the strained cyclopropane ring cleaving the C13–C14 bond in a matter of a few hours. Notably, the resulting 11-membered bicyclic ring dienone (**3**), whose structure was confirmed by X-ray diffraction (Figure 7, for the cif file, see SI Section 3), was obtained in quantitative yield. To the best of our knowledge, this is the first example of a dienone where each double bond resides on a CH_2_ bridgehead (a double anti-Bredt’s rule), although a related oxobicyclo [4.4.1] diene system (Tylopiol A, see Figure 5b) was recently described.^17^ An interesting UV spectral absorption at 285 nm (ε = 2.2 × 10^3^) was also measured for **3**, representing a red shift of about 35 nm relative to reactant **1**. This value reflects the partial through-space interaction of the two double bonds characteristic to all compounds of this type (see below).

**Figure 8:**
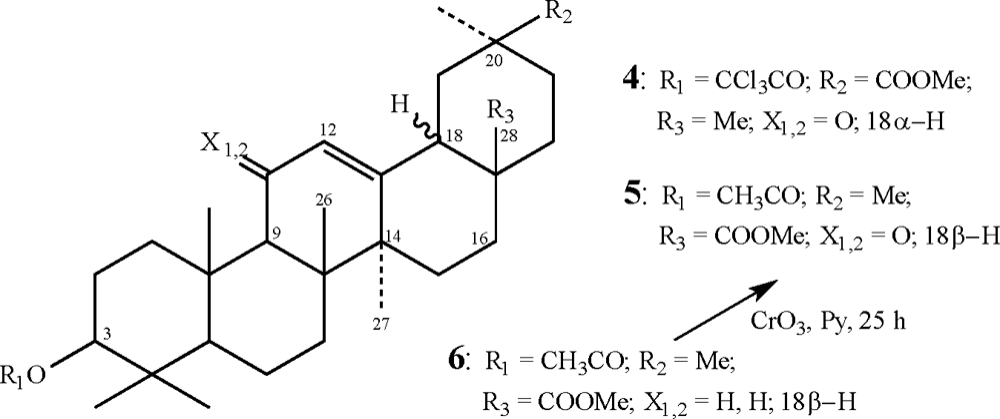
Structures of compounds **4**, **5**, and **6**. Note that **6** was oxidized to **5** with the aid of CrO_3_.

The two unique reactions described above that stem from the short-lived nature of the α-fluorocarbocation moiety are not limited to 18β-glycyrrhetic acid. To demonstrate the viability of these reactions in other triterpenoids, we considered two additional molecules. The first was methyl 3-trichloroacetoxy-α-glycyrrhetate (**4**) (rings D/E being in *trans*-configuration), and the second was methyl 3-acetoxy-11-oxooleanoate (**5**) made through oxidation of methyl 3-acetoxy-oleanoate (**6**) by chrome-trioxide (see Figure 8).^18^

**Figure 9:**
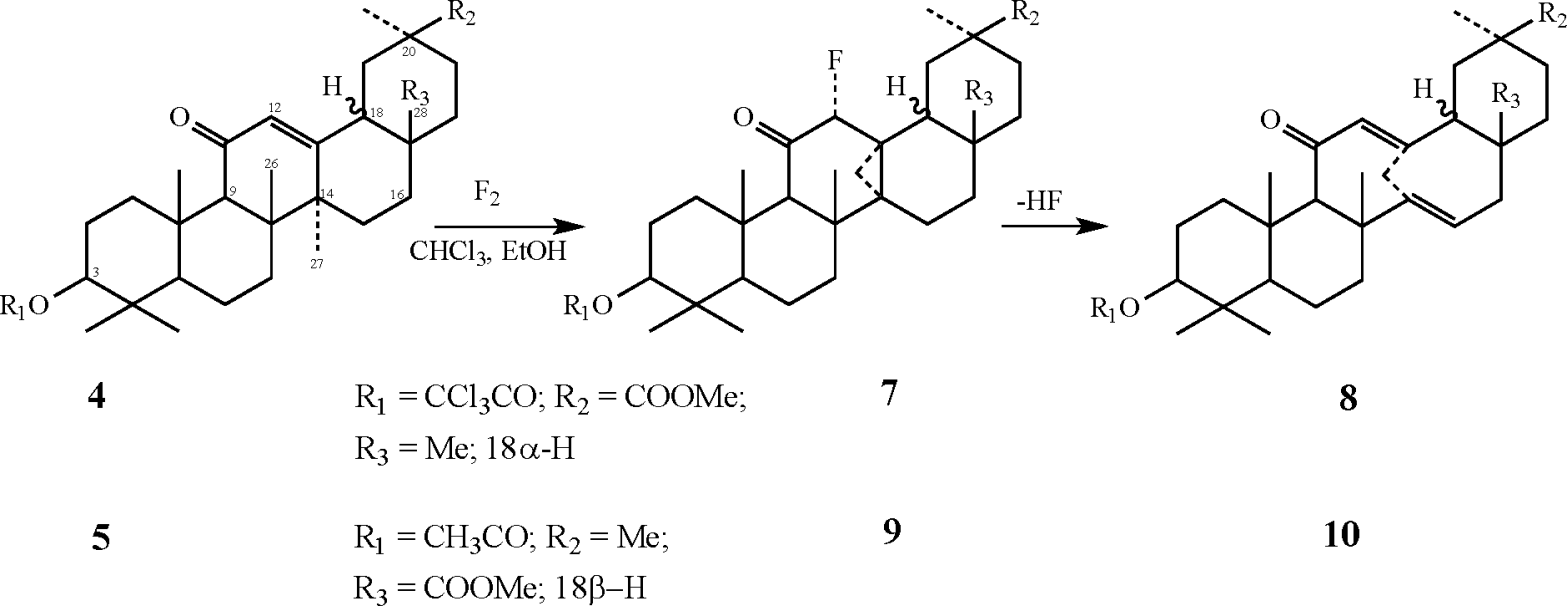
Cyclopropanation reaction course of **4** and **5** with fluorine and the consecutive dehydrofluorination.

The purpose of the fluorination of **4** was to see if the *trans*-configuration of the D/E rings affects the approach of the F_2_ molecule to the C12–C13 double bond compared to the methyl 3-acetoxy-β-glycyrrhetate (**1**). The results show that the relative arrangement of these rings has a negligible effect on the reaction, and only the angular methyl groups, which direct the attack from the α-side, govern the approach of the fluorine toward the enone. The resulting fluoro-cyclopropane derivative **7** is formed in 95% yield (Figure 9). The properties of this compound were found to be very similar to **2**, including the ^1^H NMR spectral line of one of the hydrogens in the cyclopropane ring that appeared at 0.47 ppm. Similar to the β-glycyrrhetate series, compound **7** was found to readily decompose (within a few hours), when solvents with somewhat acidic hydrogen (e.g., CHCl_3_) were present, to form the bridgehead 11-membered bicyclic ring dienone (**8**) in quantitative yields. Similar to compound **3**, this product revealed a typical UV absorption of 287 nm (ε = 3.7 × 10^3^), and the final proof was again obtained by X-ray crystallography (for the appropriate cif file see SI Section 3). To check whether the location of the carboxylic moiety has some effect on the α-fluorocarbocation, we tested also the oleanolic acid derivative **5**. The changed location of the carboxylic acid did not affect the course of the reaction. When a dilute 10% F_2_ in N_2_ was bubbled through a solution of **5** in CHCl_3_/CFCl_3_/EtOH - 1:1:0.2 at −75 °C, the fluoro-cyclopropane derivative **9** was formed in 90% yield. In turn, this compound was also converted to the dienone **10** in higher than 95% yield with a typical UV absorption of 290 nm (see Figure 9).

## Conclusions

Our findings demonstrate that the α-fluorocarbocation resulting from the addition of F–X (X = F, OAc, OTFA, and the like) to double bonds is a unique moiety, quite distinct from common carbocations. Its short life span is responsible for some unexpected reactions, such as the *syn*-addition across double bonds. When, however, the reaction’s transition state does not favor a parallel approach to the double bond as in the cases described herein, the very short living α-fluorocarbocation intermediate may be responsible for unconventional results such as a nucleophilic attack on it by a methyl group promoting unprecedented cyclopropanation. In addition, the decomposition of the strained cyclopropane expands Bredt’s rule by forming an 11-membered bicyclic ring that includes two bridgehead double bonds within a triterpene skeleton. These important findings may therefore shed light on new processes involving short-lived carbocations within the realm of fluorine chemistry and beyond.

## Experimental Section

IR spectra were recorded with an FTIR ATR spectrometer (TENSOR 27 by Bruker), and MS were measured under ESI and APPI conditions. ^1^H NMR spectra were recorded using a 400.2 MHz spectrometer with CDCl_3_ as a solvent and Me_4_Si as an internal standard. The ^19^F NMR spectra were measured at 376.5 MHz with CFCl_3_, serving as an internal standard. The proton broadband decoupled ^13^C{^1^H} NMR spectra were recorded at 100.6 MHz. NMR spectra of the various compounds studied herein are provided in SI Section 1.

### Fluorination

Fluorine is a strong oxidant and corrosive material. In organic chemistry, it is used after dilution with nitrogen or helium (generally from 1% to 20% depending on the reaction type). Such dilution can be achieved by using either an appropriate copper or Monel vacuum line constructed in a well-ventilated area or by purchasing prediluted fluorine. A detailed description of a simple setup for fluorine dilution was previously presented.^19^ The reactions themselves are carried out in standard glassware. If elementary precautions are taken, work with F_2_ (which is less toxic and less dangerous than chlorine or bromine (!)^20^) proceeds smoothly, and we have had no bad experience working with it. The reactions were usually carried out on scales of 1–5 mmol of the α,β-unsaturated carbonyl compounds, monitored by TLC or NMR. Fluorine, at concentrations of 7–10% in N_2_, was slowly passed through a cold (−78 °C) and vigorously stirred solution of the triterpene dissolved in 100 mL of CFCl_3_, 125 mL CHCl_3_, and 25 mL of EtOH. An efficient mixing is achieved by using a vibromixer, which also ensures a fine dispersion of the gas bubbles. The reactions were completed within 3–4 h. They were terminated by pouring them into 200 mL of water, washing the organic layer with NaHCO_3_ solution followed by water until neutral, drying the organic layer over MgSO_4_, and finally evaporating the solvent. The crude product was usually purified by recrystallization from EtOAc:petroleum ether. Dehydrofluorination (HF elimination) was achieved simply by stirring the fluorinated product overnight in chloroform at room temperature.

#### Methyl-3-acetoxy-12α–fluoro-13α,14α-cyclopropane-β-glycyrrhetate (**2**)

**2** was prepared from methyl-3-acetoxy-β-glycyrrhetate (**1**) (0.97 g, 1.8 mmol) as described above. A white solid, mp 285–286 °C, (0.90 g, 90% yield) was obtained; ^1^H NMR (400 MHz, CDCl_3_) δ 4.53 (d, *J* = 48.8 Hz, 1 H), 4.48 (dd, *J*_1_ = 10.7, J_2_ = 5.7 Hz, 1 H), 3.68 (s, 3 H), 2.46 (dt, *J*_1_ = 13.7, *J*_2_ = 3.6 Hz, 1 H), 2.16–2.13 (m, 1 H), 2.04 (s, 3 H), 1.92–1.60 (m, 9 H), 1.46–1.27 (m, 6 H), 1.16 (s, 3 H), 1.15 (s, 3 H), 1.14 (s, 3 H), 1.13–0.75 (m, 5 H), 0.86 (s, 3 H), 0.85 (s, 6 H), 0.45 (d, *J* = 6.7 Hz, 1 H, one of the two cyclopropane hydrogens) ppm; ^13^C{^1^H} NMR (100 MHz, CDCl_3_) δ 203.8 (d, *J* = 14.6 Hz), 177.8, 171.0, 97.7 (d, *J* = 183.3 Hz), 80.4, 63.0, 55.2, 51.8, 43.5, 42.0, 40.2, 38.4, 38.1, 38.0, 37.9, 37.6, 37.0, 34.2 (d, *J* = 8.8 Hz), 31.1, 29.9, 29.6, 28.2, 28.1, 27.9, 26.3, 23.5, 21.4, 21.2, 20.6 (d, *J* = 2.5 Hz), 17.8, 17.0, 16.7, 11.3 (d, *J* = 8.4 Hz) ppm; ^19^F NMR (376 MHz, CDCl_3_, CFCl_3_-int standard) δ −176.9 (d, *J* = 48.9 Hz) ppm; HRMS (ESI-TOF) *m*/*z* [M + Na]^+^ Calcd for C_33_H_49_FO_5_Na, 567.3462; Found, 567.3463; Anal. Calcd for C_33_H_49_FO_5_: C, 72.76; H, 9.07. Found: C, 72.34; H, 9.07; IR 1727, 1711 cm^–1^.

#### Bicyclic **3** Prepared by Dehydrofluorination of **2**

**3** was prepared as described above. A white solid, mp 230–232 °C, (0.87 g, >95% yield) was obtained; ^1^H NMR (400 MHz, CDCl_3_) δ 5.69 (s, 1 H), 5.22 (t, *J* = 7.0 Hz, 1 H), 4.50 (dd, *J*_1_ = 11.8, *J*_2_ = 4.7 Hz, 1 H), 3.67 (s, 3 H), 3.36 (d, *J* = 13.1 Hz, 1 H), 3.11 (d, *J* = 13.2 Hz, 1 H), 2.95 (dd, *J*_1_ = 14.4, *J*_2_ = 6.2 Hz, 1 H), 2.73 (s, 1 H), 2.26–2.06 (m, 4 H), 1.89–1.57 (m, 7 H), 1.49–1.28 (m, 6 H), 1.42 (s, 3 H), 1.25 (s, 3 H), 1.12 (s, 3 H), 1.01 (dt, *J*_1_ = 13.1, *J*_2_ = 3.4 Hz, 1 H), 0.91–0.80 (m, 61 2 H), 0.89 (s, 3 H), 0.86 (s, 3 H), 0.71 (s, 3 H) ppm; ^13^C{^1^H} NMR (100 MHz, CDCl_3_) δ 199.5, 177.3, 171.1, 159.8, 151.3, 129.9, 119.3, 80.8, 67.8, 56.3, 54.7, 52.0, 43.4, 39.9, 39.5, 39.2, 39.1, 38.1, 38.0, 37.9, 35.1, 33.8, 31.9, 29.8, 28.7, 28.2, 28.0, 23.6, 21.4, 20.6, 18.8, 17.9, 16.7 ppm; HRMS (APPI) *m*/*z* [M + H]^+^ Calcd for C_33_H_49_O_5_, 525.3580; Found, 525.3579; Anal. Calcd for C_33_H_48_O_5_: C, 75.53; H, 9.22. Found: C, 75.21; H, 9.08; IR 1730, 1639 cm-1; UV (DCM) λ_max_, nm (ε): 285 (2195).

#### Methyl-3-trichloroacetoxy-α-glycyrrhetate (**4**)

**4** was prepared by suspending methyl-18α- glycyrrhetic acid (0.23 g, 0.5 mmol) in dichloromethane (30 mL), triethylamine (0.13 mL, 0.9 mmol), tricholoacetic anhydride (0.22 mL, 1.2 mmol) and DMAP (cat.). The mixture was stirred overnight and then quenched by diluted HCl (2M). The phases were separated, the organic phase dried over Na_2_SO_4_ and the solvent removed in vacuo. The product was recrystallized from PE/EtOAc. A white solid, mp 293–295 °C, (0.3 g, >95% yield) was obtained; ^1^H NMR (400 MHz, CDCl_3_) δ 5.58 (s, 1 H), 4.64 (dd, *J*_1_ = 11.9, *J*_2_ = 4.7 Hz, 1 H), 3.69 (s, 3 H), 2.79 (dt, *J*_1_ = 13.7, *J*_2_ = 3.6 Hz, 1 H), 2.29 (s, 1 H), 2.25–2.22 (m, 1 H), 2.02–1.01 (m, 17 H), 1.35 (s, 3 H), 1.24 (s, 3 H), 1.22 (s, 3 H), 1.14 (s, 3 H), 0.97 (s, 6 H), 0.83 (d, *J* = 10.2 Hz, 1H), 0.72 (s, 3 H) ppm; ^13^C{^1^H} NMR (100 MHz, CDCl_3_) δ 199.5, 178.9, 166.0, 161.8, 124.3, 87.2, 60.7, 55.2, 52.1, 45.1, 44.0, 42.7, 40.6, 38.8, 38.7, 37.8, 36.9, 36.1, 35.7, 33.8, 32.0, 29.9, 28.6, 28.2, 26.9, 23.0, 21.0, 20.9, 18.7, 17.6, 16.8, 16.2 ppm; HRMS (ESI-TOF) *m*/*z* [M + Na]^+^ Calcd for C_33_H_47_Cl_3_O_5_Na 651.2387; Found, 651.2389; IR 1760, 1728, 1651 cm^–1^.

#### Methyl-3-trichloroacetoxy-12α-fluoro-13α,14α-cyclopropane-α-glycyrrhetate (**7**)

**7** was prepared from (**4**) (0.30 g, 0.5 mmol) as described above. A white solid, mp >265 °C dec., (0.29 g, 95% yield) was obtained; ^1^H NMR (400 MHz, CDCl_3_) δ 4.60 (dd, *J*_1_ = 11.8, *J*_2_ = 4.9 Hz, 1 H), 4.55 (d, *J* = 47.7, 1 H), 3.69 (s, 3 H), 2.37 (dt, *J*_1_ = 13.7, *J*_1_ = 3.6 Hz, 1 H), 2.08 (s, 1 H), 2.00–0.79 (m, 20 H), 1.23 (s, 6H), 1.12 (s, 3 H), 0.95 (s, 6 H), 0.76 (s, 3 H), 0.47 (d, J = 6.2 Hz, 1 H, one of the two cyclopropane hydrogens) ppm; ^13^C{^1^H} NMR (100 MHz, CDCl_3_) δ 203.1 (d, *J* = 15.8 Hz), 179.1, 161.7, 88.3 (d, *J* = 178.6 Hz), 86.8, 61.4, 55.0, 52.1, 44.2, 43.2, 41.3, 38.6, 37.7, 37.6, 36.3, 36.0, 35.5 (d, *J* = 9.3 Hz), 32.4, 31.6, 29.8, 29.1, 28.0, 27.1, 26.9, 22.7, 21.0, 20.9, 20.7 (d, *J* = 10.9 Hz), 19.9, 17.8, 16.8, 16.5, 15.2 ppm; ^19^F NMR (376 MHz, CDCl_3_, CFCl_3_-int standard) δ −166.8 (d, *J* = 48.1 Hz) ppm; HRMS (ESI-TOF) *m*/*z* [M + Na]^+^ Calcd for C_33_H_46_Cl_3_FO_5_Na, 669.2293; Found, 669.2289; IR 1753, 1736, 1715 cm^–1^.

#### Bicyclic **8** Prepared by Dehydrofluorination of **7**

**8** was prepared as described above. A white solid, mp >277 °C dec., (0.28 g, >95% yield) was obtained; ^1^H NMR (400 MHz, CDCl_3_) δ 5.49 (s, 1 H), 5.37 (t, *J* = 7.1 Hz, 1 H), 4.63 (dd, *J*_1_ = 11.9, *J*_2_ = 4.7 Hz, 1 H), 3.68 (s, 3 H), 3.54 (d, *J* = 13.0 Hz, 1 H), 3.12 (d, *J* = 13.0 Hz, 1 H), 2.73 (s, 1 H), 2.48 (dd, *J*_1_ = 14.2, *J*_2_ = 6.4 Hz, 1 H), 2.26 (dt, *J*_1_ = 13.4, *J*_2_ = 3.6 Hz, 1 H), 2.02–0.92 (m, 15 H), 2.05 (s, 3 H), 1.98–0.97 (m, 15 H), 1.46 (s, 3 H), 1.31 (s, 3 H), 1.14 (s, 3 H), 0.99 (s, 3 H), 0.96 (s, 3 H), 0.60 (s, 3 H) ppm; ^13^C{^1^H} NMR (100 MHz, CDCl_3_) δ 199.1, 178.7, 161.8, 161.2, 151.5, 126.4, 119.6, 87.2, 67.8, 56.2, 54.5, 52.1, 43.4, 42.0, 41.8, 39.8, 39.1, 38.7, 38.2, 37.9, 36.6, 31.6, 29.8, 29.4, 28.1, 22.9, 21.3, 63 20.6, 18.8, 18.0, 16.5, 15.2 ppm; HRMS (ESI-TOF) *m*/*z* [M + Na]^+^ Calcd for C_33_H_45_Cl_3_O_5_Na, 649.2230; Found, 649.2232; IR 1759, 1725, 1638 cm^–1^; λ_max_, nm (ε): 287(3698).

#### Methyl-3β-acetoxy-12α-fluoro-13α,14α-cyclopropane-oleanolate (**9**)

**9** was prepared from methyl 3β-acetoxy-11-oxo-oleanolate (**5**) (0.67 g, 1.3 mmol) as described above. A white solid, mp 123–125 °C, (0.60 g, 85% yield) was obtained; ^1^H NMR (400 MHz, CDCl_3_) δ 4.82 (d, *J* = 48.5 Hz, 1 H), 4.47 (dd, *J*_1_ = 10.5, *J*_2_ = 6.1 Hz, 1 H), 3.66 (s, 3 H), 2.77 (dd, *J*_1_ = 12.8, *J*_2_ = 4.6 Hz, 1 H), 2.59 (dt, *J*_1_ = 13.6, *J*_2_ = 3.6 Hz, 1 H), 2.03 (s, 3 H), 1.92–0.73 (m, 20 H), 1.10 (s, 3 H), 0.94 (s, 3 H), 0.91 (s, 3 H), 0.88 (s, 3 H), 0.86 (s, 3 H), 0.85 (s, 3 H), 0.84 (s, 3 H), 0.45 (d, *J* = 6.7 Hz, 1 H, one of the two cyclopropane hydrogens) ppm; ^13^C{^1^H} NMR (100 MHz, CDCl_3_) δ 204.0 (d, J = 13.4 Hz), 178.0, 171.0, 96.9 (d, *J* = 180.9 Hz), 80.4, 63.7, 55.3, 52.0, 44.8, 41.2, 38.6, 38.3, 38.1, 37.5, 37.3, 34.5, 34.0, 33.7 (d, *J* = 9.1 Hz), 32.9, 32.6, 30.3, 28.1, 24.0, 23.5, 22.6, 22.0, 21.4, 20.9, 17.7, 16.8, 16.7, 14.2, 12.3 (d, *J* = 7.0 Hz) ppm; ^19^F NMR (376 MHz, CDCl_3_, CFCl_3_-int standard) δ −180.8 (d, *J* = 48.6 Hz) ppm; HRMS (ESI-TOF) *m*/*z* [M + Na]^+^ Calcd for C_33_H_49_FO_5_Na, 567.3462; Found, 567.3466; IR 1722 cm^–1^.

#### Bicyclic **10** Prepared by Dehydrofluorination of **9**

**10** was prepared using the dehydrofluorination procedure described above. A white solid, mp 219–221 °C, (0.58 g, >95% yield) was obtained; ^1^H NMR 5.68 (s, 1 H), 5.38 (t, *J* = 6.7 Hz, 1 H), 4.49 (dd, *J*_1_ = 11.7, *J*_2_ = 4.8 Hz, 1 H), 3.59 (s, 3 H), 3.44 (dd, *J*_1_ = 13.1, *J*_2_ = 4.0 Hz, 1 H), 3.38 (d, *J* = 13.1 Hz, 1 H), 3.03 (d, *J* = 14.1 Hz, 1 H), 3.01(d, *J* = 14.3 Hz, 1 H), 2.68 (s, 1 H), 2.20–2.13 (m, 2 H), 2.04 (s, 3H), 1.95–1.83 (m, 2 H), 1.78–1.24 (m, 12 H), 1.39 (s, 3 H), 1.04 (s, 3 H), 1.05 (s, 3 H), 1.00 (s, 3 H), 0.89 (s, 3 H), 0.85 (s, 3 H) ppm; ^13^C{^1^H} NMR (100 MHz, CDCl_3_) δ 199.3, 176.4, 171.1, 163.0, 153.9, 130.9, 120.5, 80.8, 68.3, 56.3, 54.7, 51.8, 46.7, 39.2, 38.2, 38.1, 38.0, 37.9, 34.7, 34.4, 33.2, 30.4, 29.9, 29.8, 28.2, 24.2, 23.6, 21.4, 21.1, 18.8, 17.9, 16.7 ppm; HRMS (ESI-TOF) *m*/*z* [M + Na]^+^ Calcd for C_33_H_48_O_5_Na, 547.3399; Found, 547.3394; Anal. Calcd for C_33_H_48_O_5_: C, 75.53; H, 9.22. Found: C, 75.25; H, 8.79; IR 1738, 1712, 1634 cm^–1^; UV (CH_3_CN) λ_max_, nm (ε): 287(2000).

## Supporting Information Available

^1^H, ^13^C, and ^19^F NMR spectra; calculated spectrum; computational details; details on X-ray cif files; and Cartesian coordinates of the calculated structures (PDF)

## Accession Codes

CCDC 2002953 and 2002977–2002979 contain the supplementary crystallographic data for this paper. These data can be obtained free of charge via www.ccdc.cam.ac.uk/data_request/cif, or by emailing data_request@ccdc.cam.ac.uk, or by contacting The Cambridge Crystallographic Data Centre, 12 Union Road, Cambridge CB2 1EZ, UK; fax: +44 1223 336033.
