# Supplementary material for: The Chemistry of Short-Lived α-Fluorocarbocations
Source: J Org Chem. 2021 Feb 22;86(5):3882–9. doi: 10.1021/acs.joc.0c02731 (PMC8023664; doi:10.1021/acs.joc.0c02731)
Supplement: Supplementary file 1 — jo0c02731_si_001.pdf [file jo0c02731_si_001.pdf]

The Chemistry of Short-Lived α-Fluorocarbocations

Supplementary Information

Shlomo Rozen,[*,a] Inna Vints,[a] Ana Lerner,[a,d] Oded Hod,[a, b] Edward N. Brothers[*,c] and Salvador Moncho[c]

[a] School of Chemistry, The Raymond and Beverly Sackler Faculty of Exact Sciences, Tel Aviv University, Tel Aviv 6997801, Israel
[b] The Sackler Center for Computational Molecular and Materials Science, Tel Aviv University, Tel Aviv 6997801, Israel
[c] Science Program, Texas A&M University at Qatar, Education City, Doha 23874, Qatar
[d] Chemistry Department, Ben-Gurion University of the Negev, Beer-Sheva 84105 (Israel)

Table of Contents

1. Measured NMR Spectra — Pages S2-S12
2. Computational — Pages S13-S23
3. Experimental X-Ray, CIF files — Pages S24
4. Cartesian coordinates of the calculated structures — Pages S25-S44

Section 1. Measured NMR Spectra

Compound 2: Methyl-3-acetoxy-12α–fluoro-13α,14α-cyclopropane-β-Glycyrrhetate ^{1}H NMR (400 MHz, $CDCl_3$)

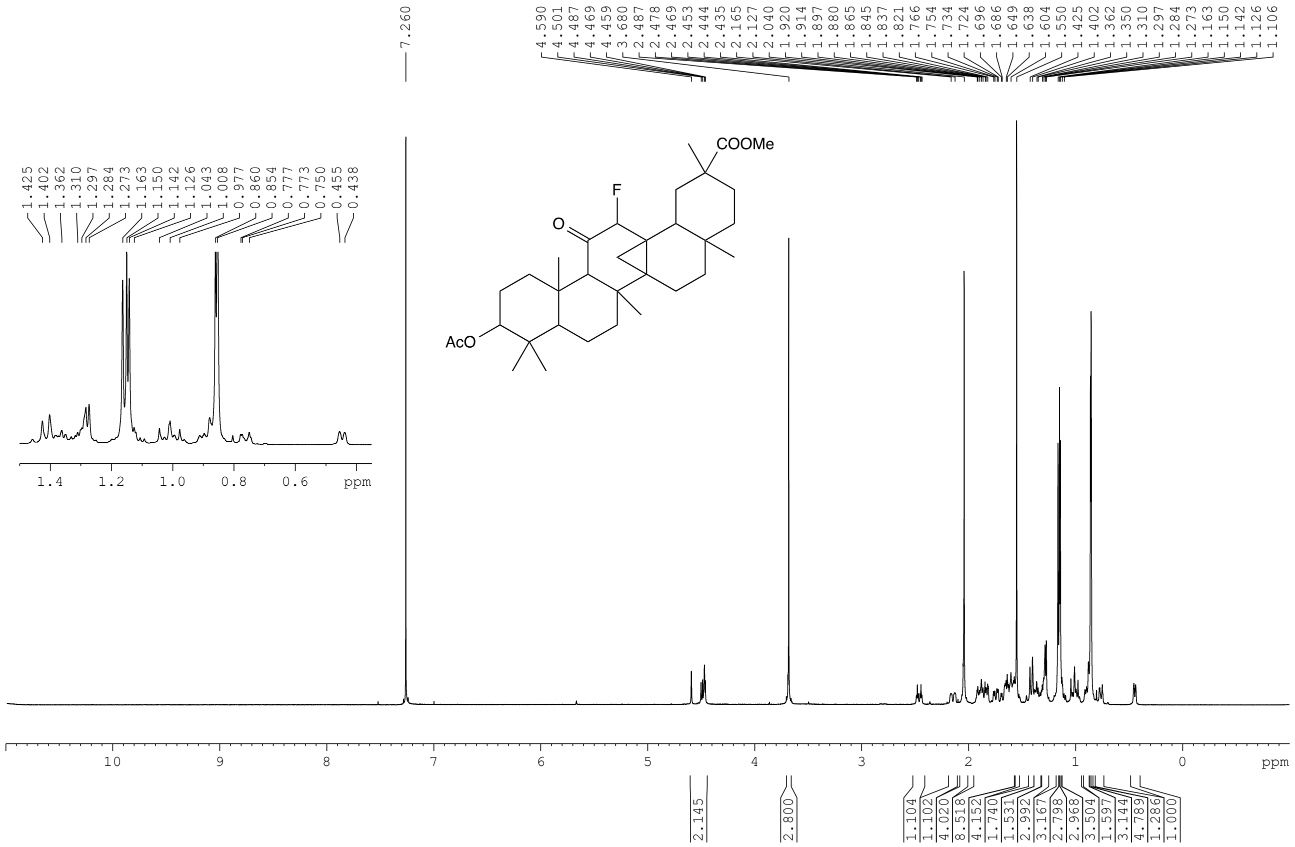

Compound 2: Methyl-3-acetoxy-12α–fluoro-13α,14α-cyclopropane-β-Glycyrrhetate $^{13}C\{1H\}$ NMR (100 MHz, $CDCl_3$)

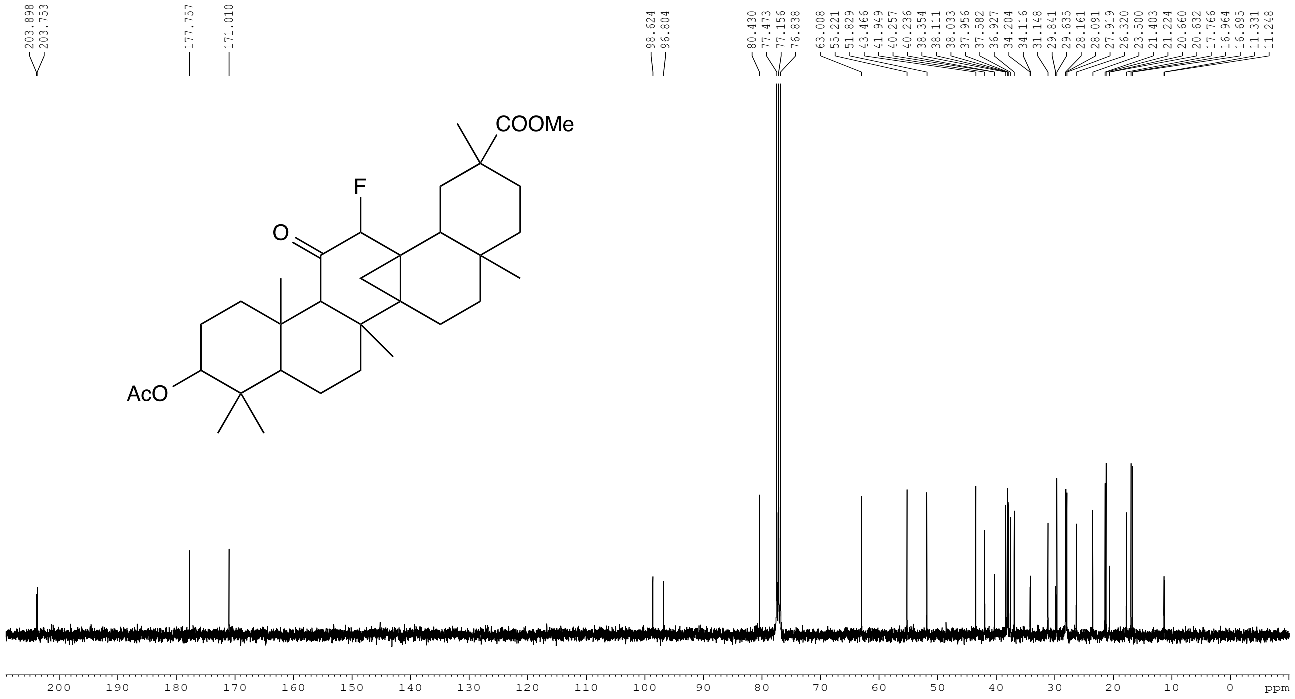

Compound 2: Methyl-3-acetoxy-12α–fluoro-13α,14α-cyclopropane-β-Glycyrrhetate ^{19}F NMR (376 MHz, $CDCl_3$, $CFCl_3$-int standard)

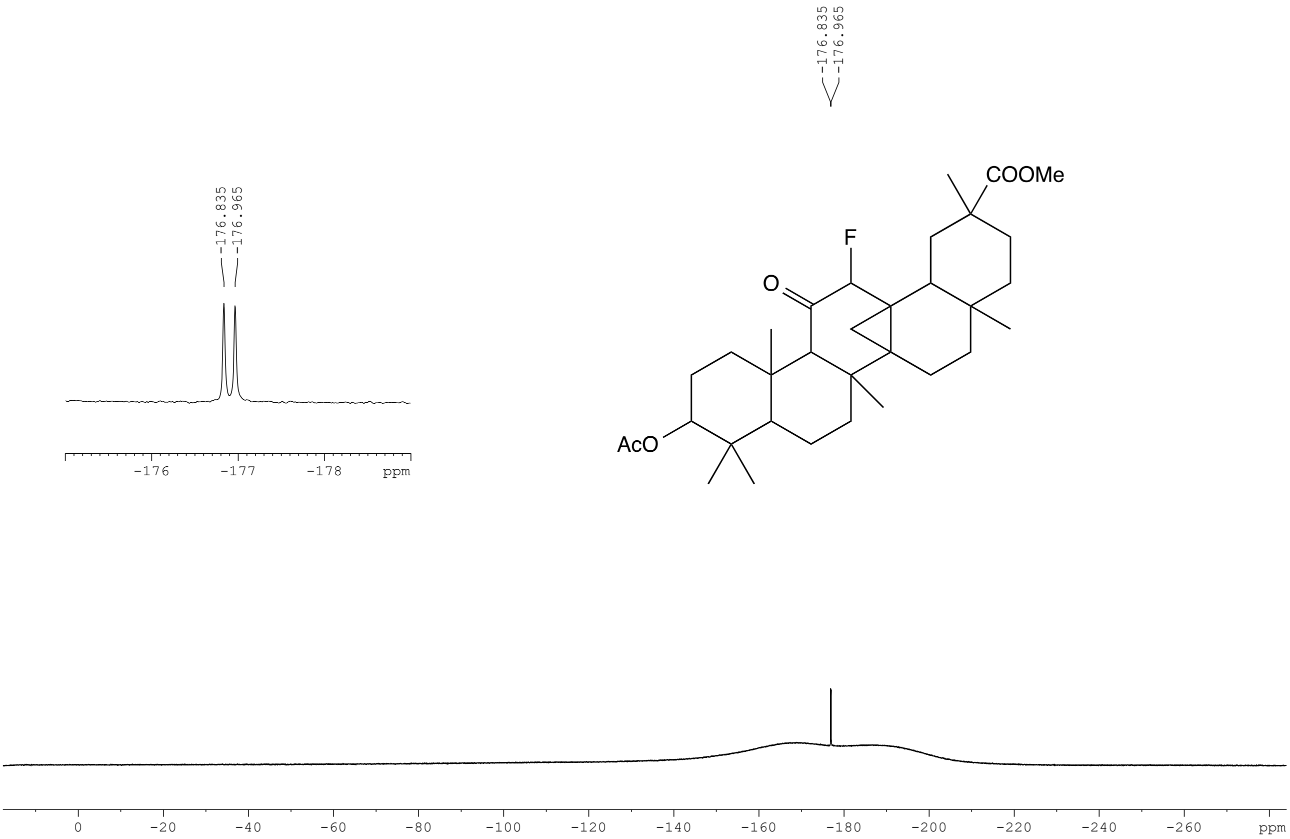

Compound 2: Methyl-3-acetoxy-12α–fluoro-13α,14α-cyclopropane-β-Glycyrrhetate ^{13}C{1H} NMR (100 MHz, $CDCl_3$) (DEPT)

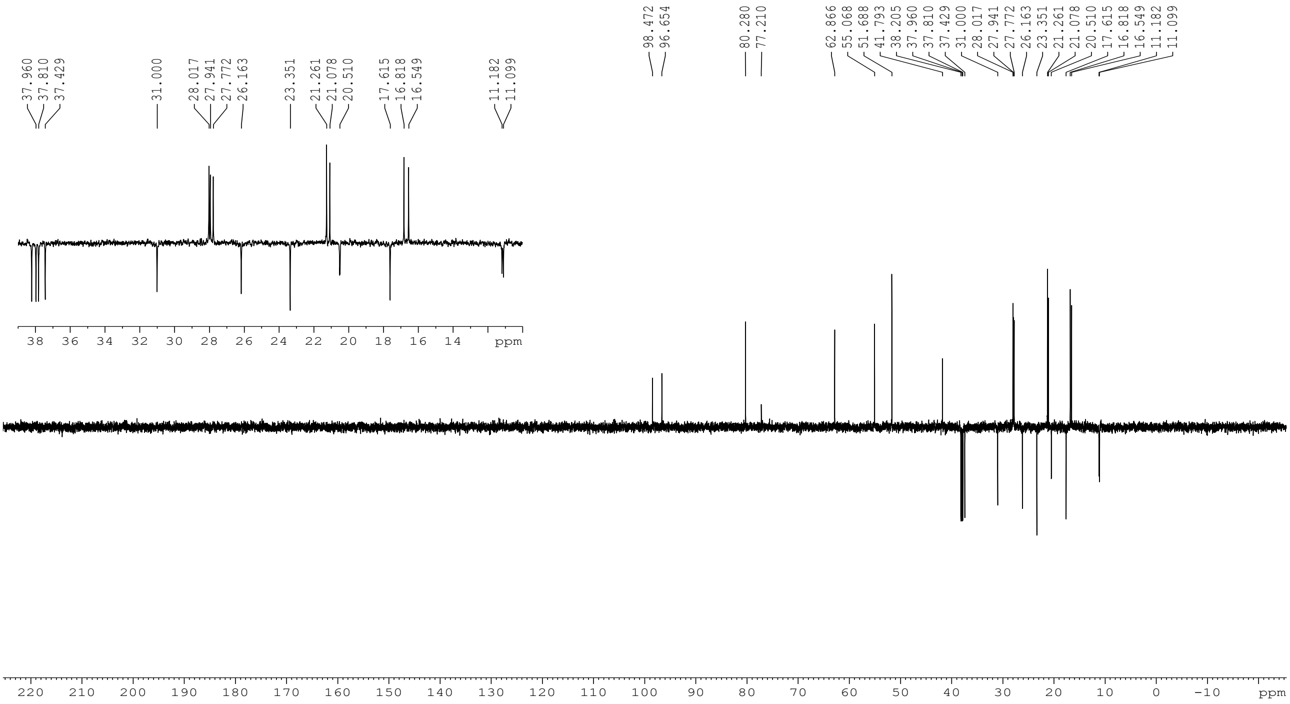

Compound 3: dehydrofluorination of 2 ^{1}H NMR (400 MHz, $CDCl_3$)

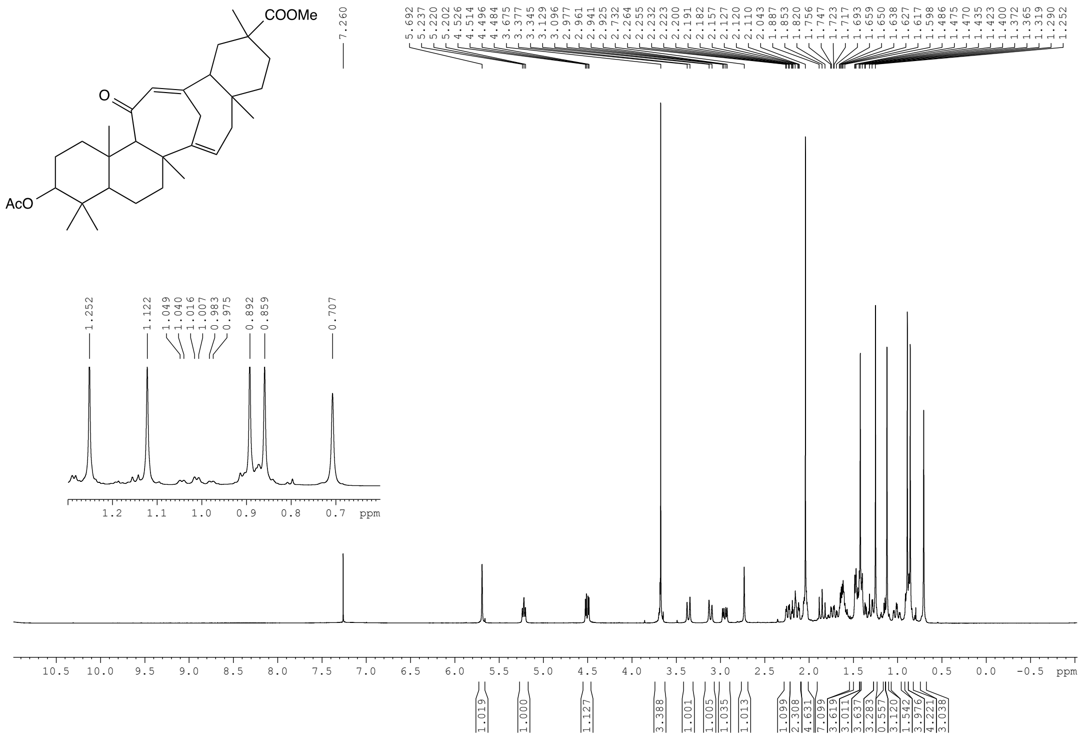

Compound 3: dehydrofluorination of 2 ^{13}C{1H} NMR (100 MHz, $CDCl_3$)

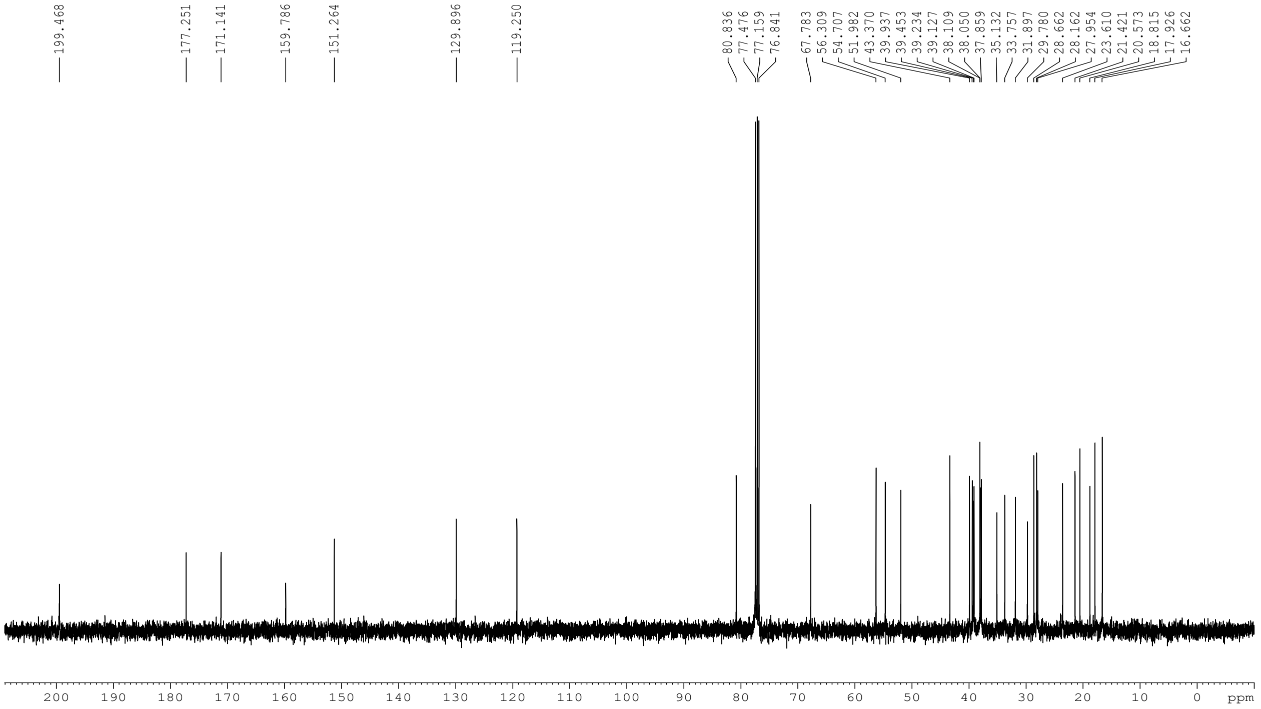

Compound 3: dehydrofluorination of 2 $^{13}C\{1H\}$ NMR (100 MHz, $CDCl_3$ (DEPT)

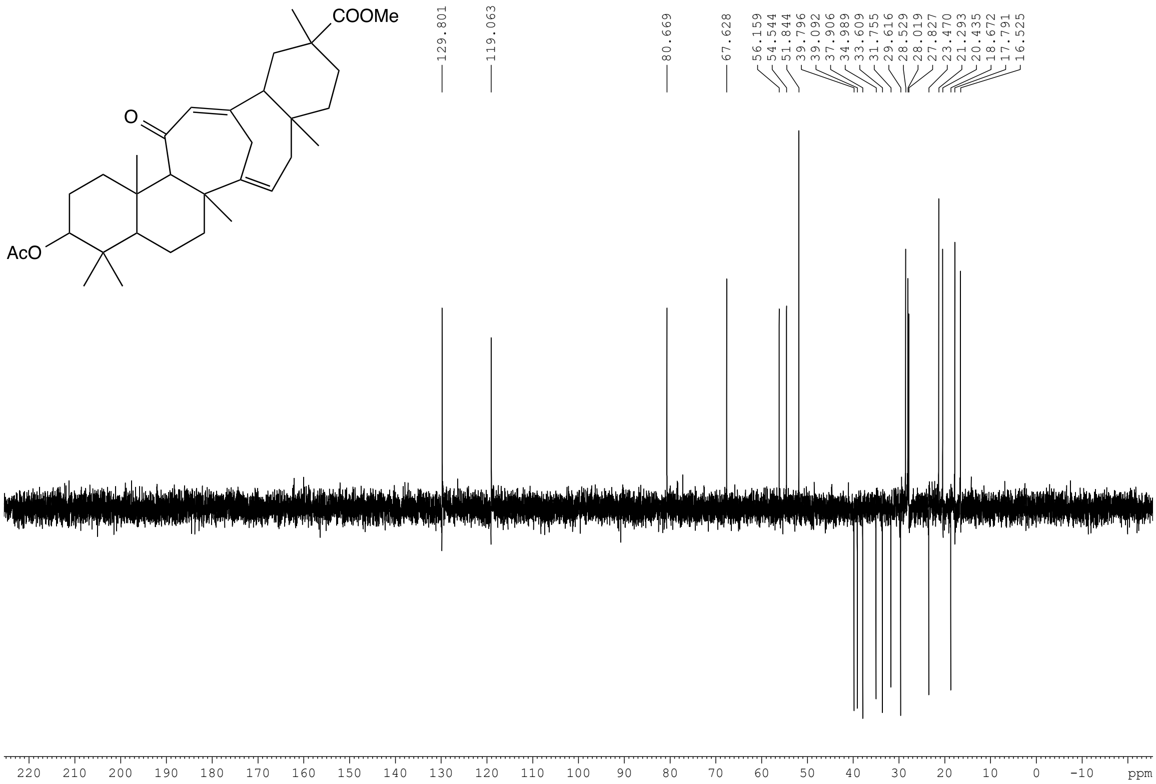

Compound 4: Methyl-3-trichloroacetoxy-α-glycyrrhetate ^1H NMR (400 MHz, $CDCl_3$)

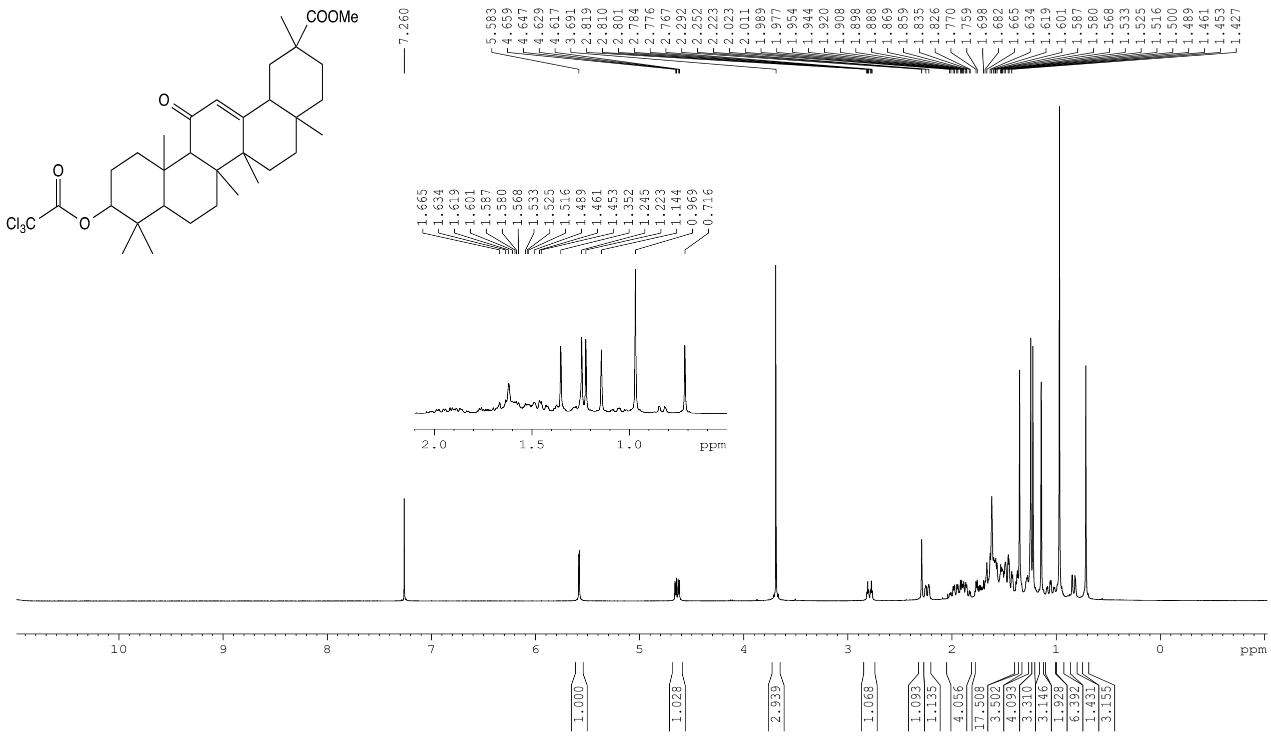

Compound 4: Methyl-3-trichloroacetoxy-α-glycyrrhetate $^{13}C\{^1H\}$ NMR (100 MHz, $CDCl_3$)

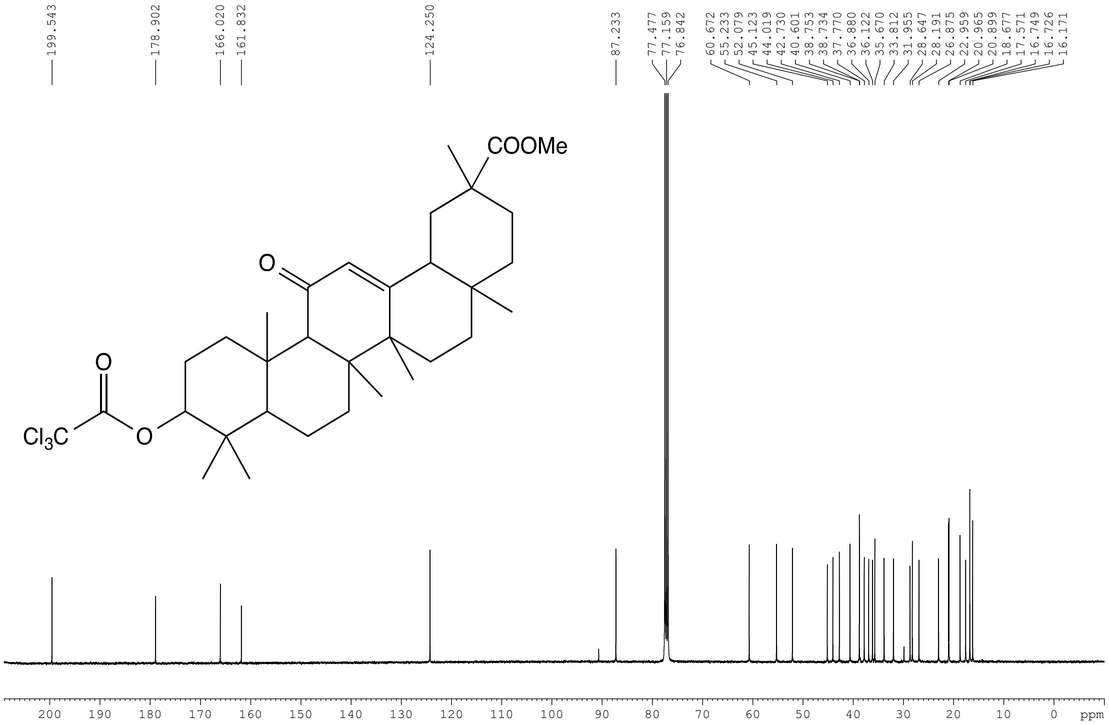

Compound 7: Methyl-3-trichloroacetoxy-12α–fluoro-13α,14α-cyclopropane-α-Glycyrrhetate ^1H NMR (400 MHz, $CDCl_3$)

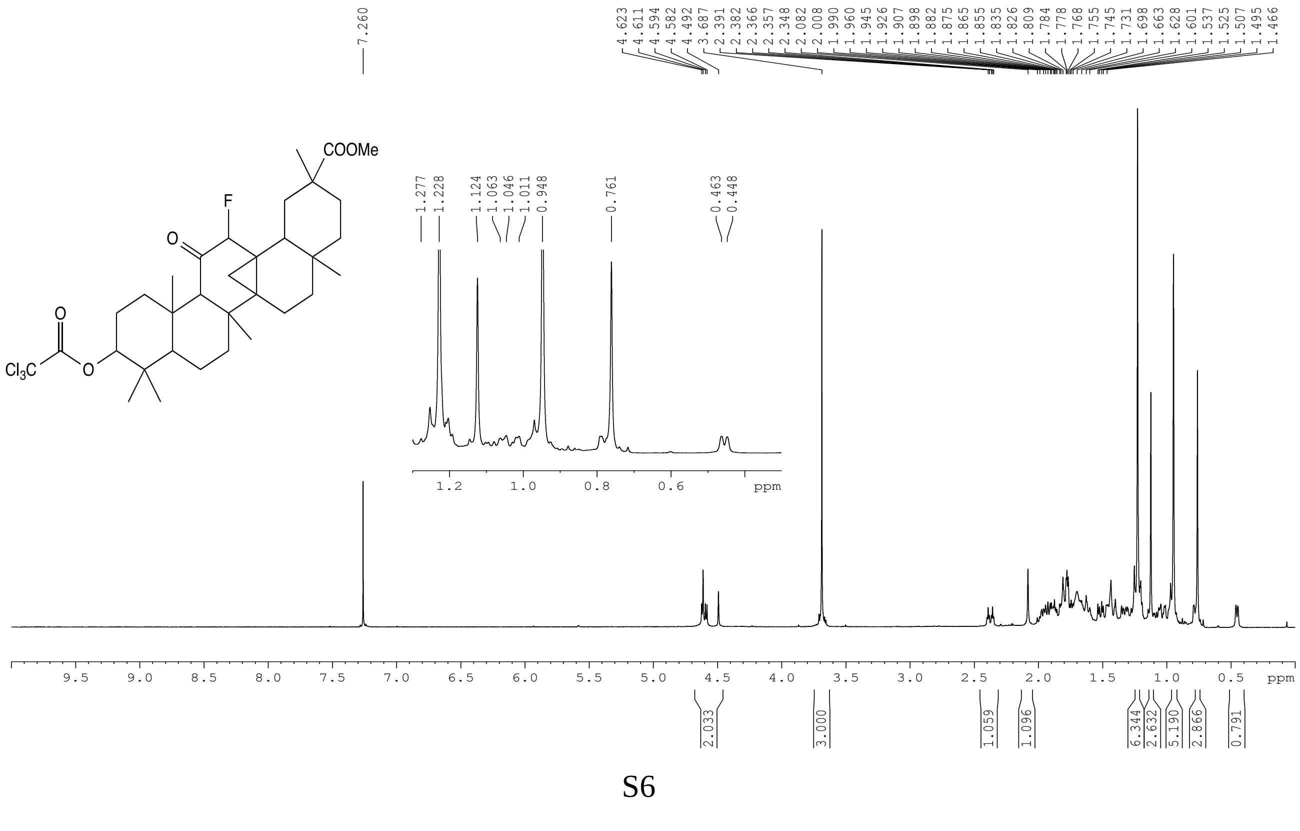

Compound 7: Methyl-3-trichloroacetoxy-12α–fluoro-13α,14α-cyclopropane-α-Glycyrrhetate $^{13}C\{^1H\}$ NMR (100 MHz, $CDCl_3$)

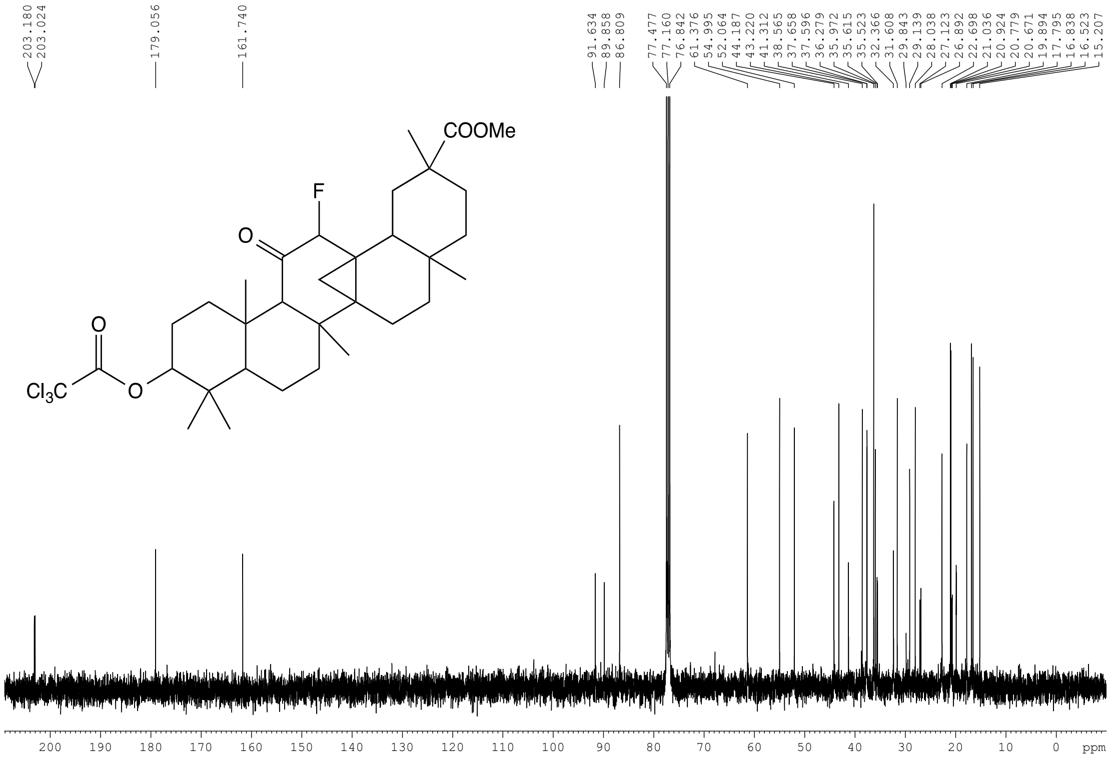

Compound 7: Methyl-3-trichloroacetoxy-12α–fluoro-13α,14α-cyclopropane-α-Glycyrrhetate ^{19}F NMR(376 MHz, $CDCl_3$, $CFCl_3$-int standard)

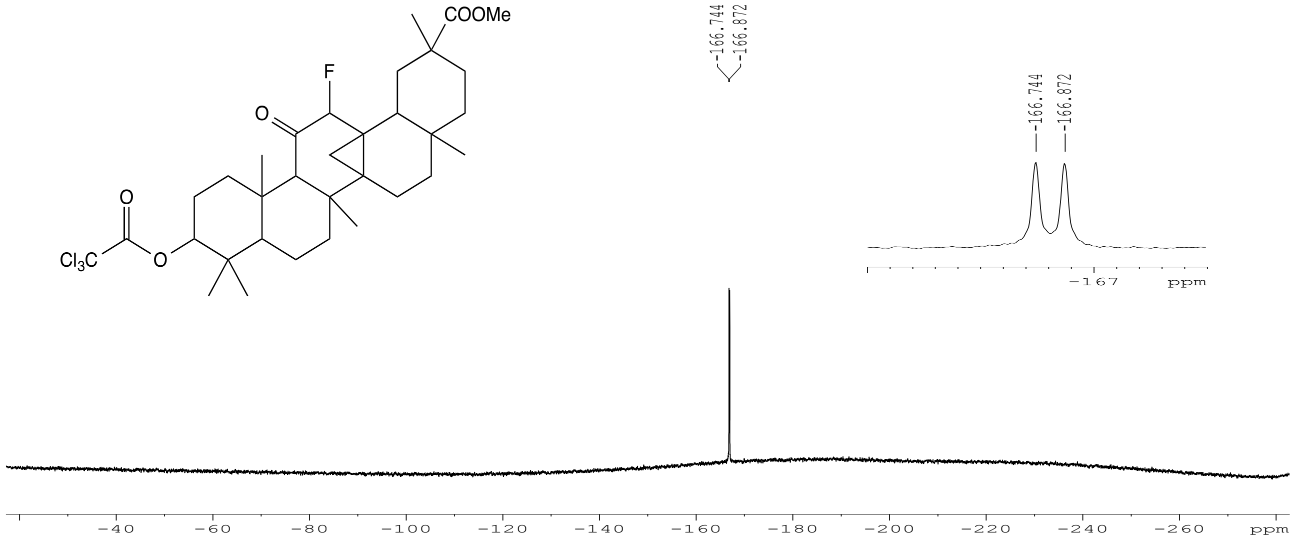

Compound 8: dehydrofluorination of 7 ^{1}H NMR (400 MHz, $CDCl_3$)

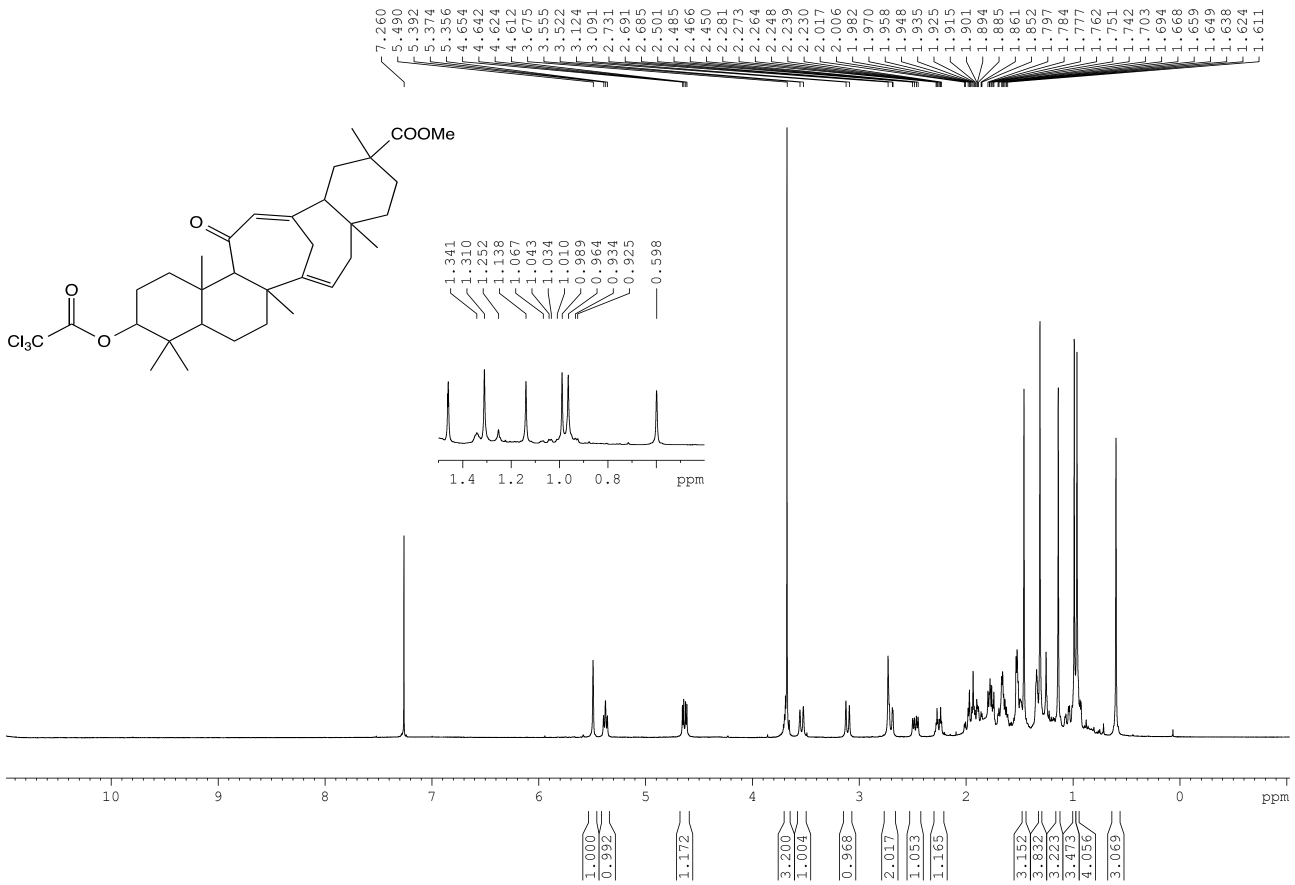

Compound 8: dehydrofluorination of 7 ^{13}C{1H} NMR (400 MHz, $CDCl_3$)

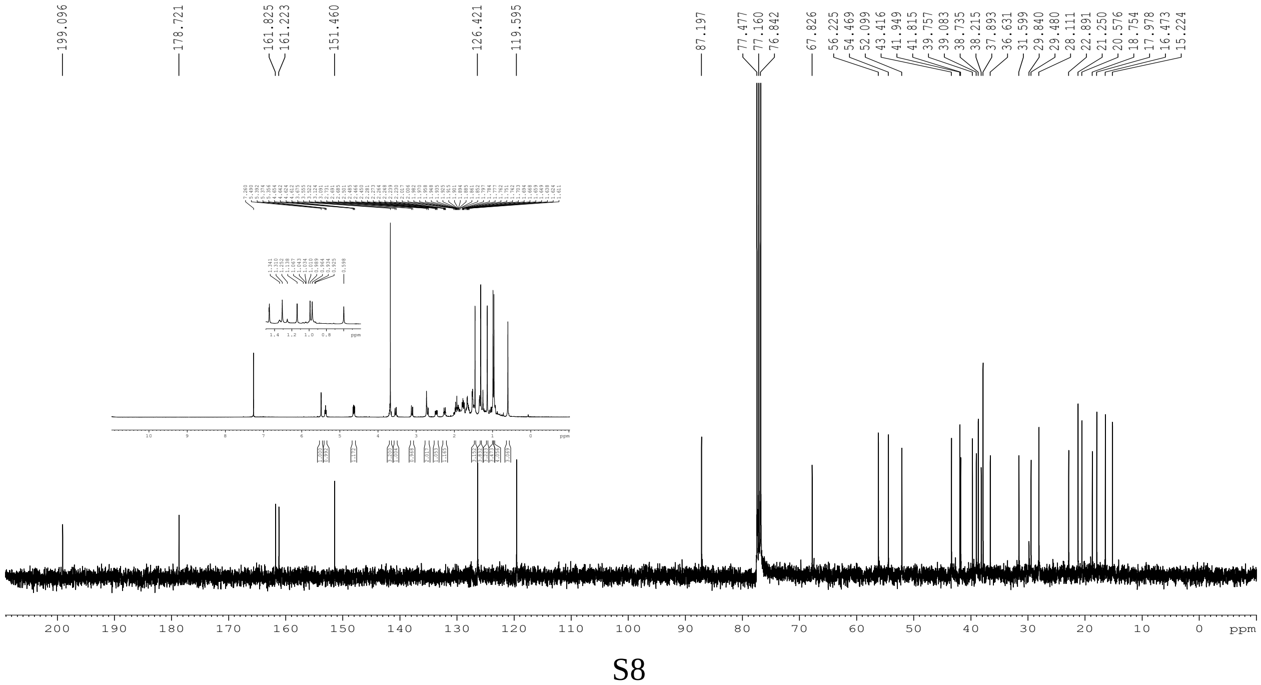

Compound 9: Methyl-3β-acetoxy-12α-fluoro-13α,14α-cyclopropane-oleanolate ^{1}H NMR (400 MHz, CDCl$_3$)

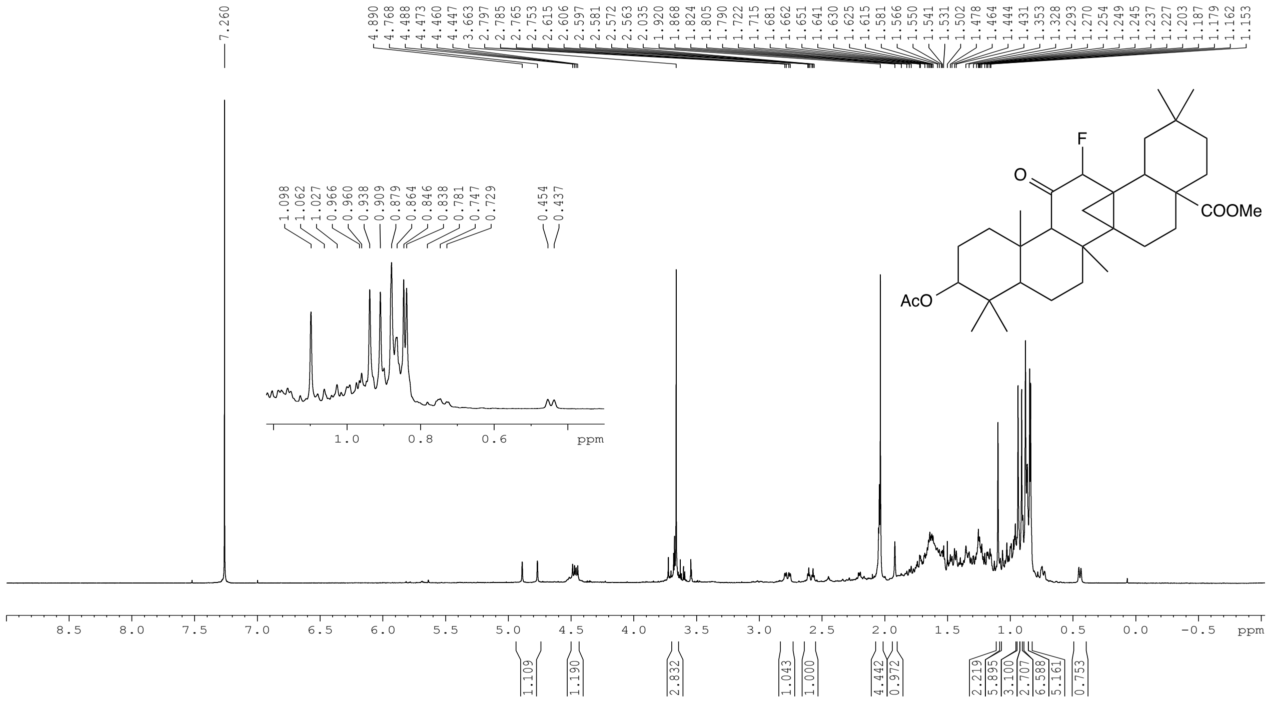

Compound 9: Methyl-3β-acetoxy-12α-fluoro-13α,14α-cyclopropane-oleanolate ^{13}C{1H} NMR (100 MHz, CDCl$_3$)

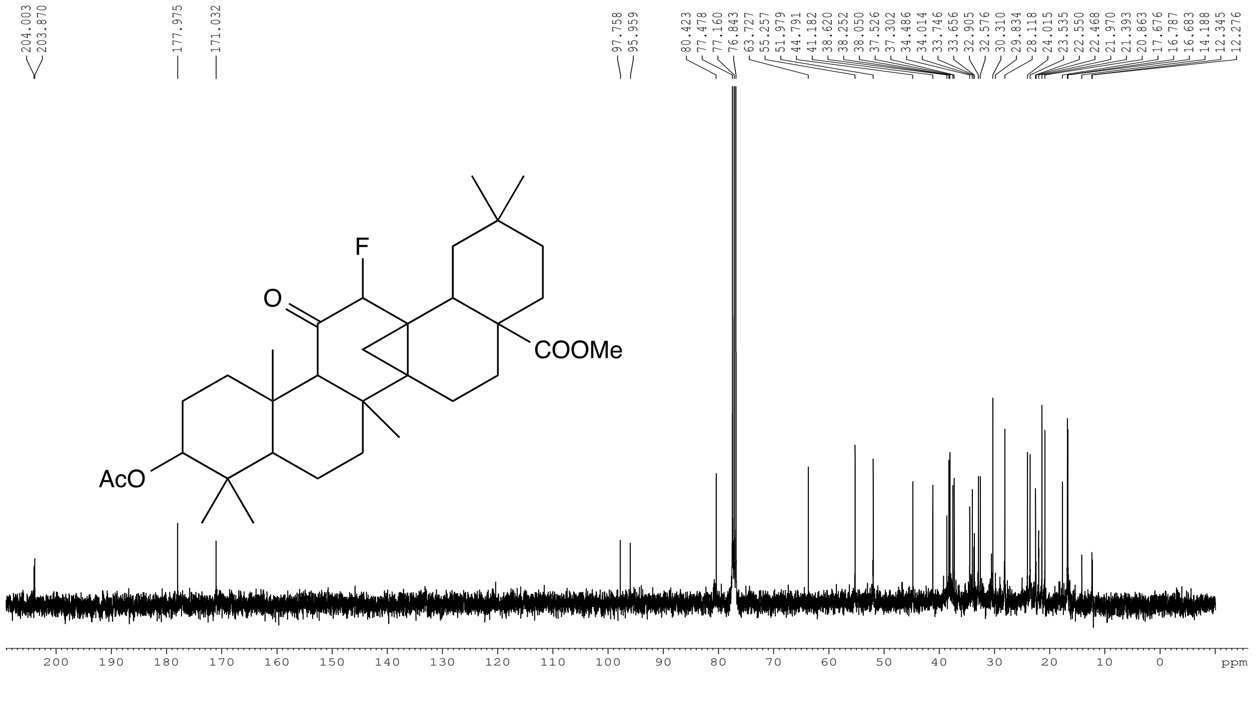

Compound 9: Methyl-3β-acetoxy-12α-fluoro-13α,14α-cyclopropane-oleanolate $^{13}C\{1H\}$ NMR (100 MHz, $CDCl_3$ (DEPT)

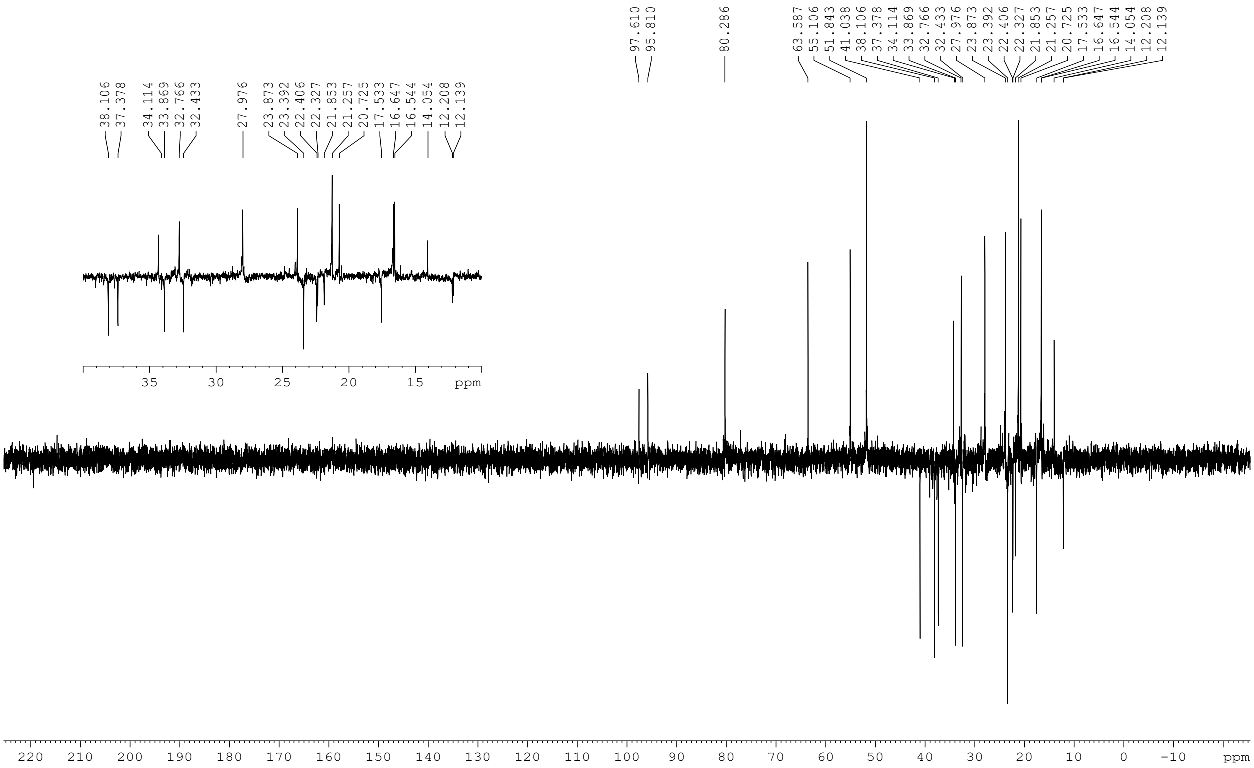

Compund 9: Methyl-3β-acetoxy-12α-fluoro-13α,14α-cyclopropane-oleanolate ^{19}F NMR (376 MHz, $CDCl_3$, $CFCl_3$-int standard)

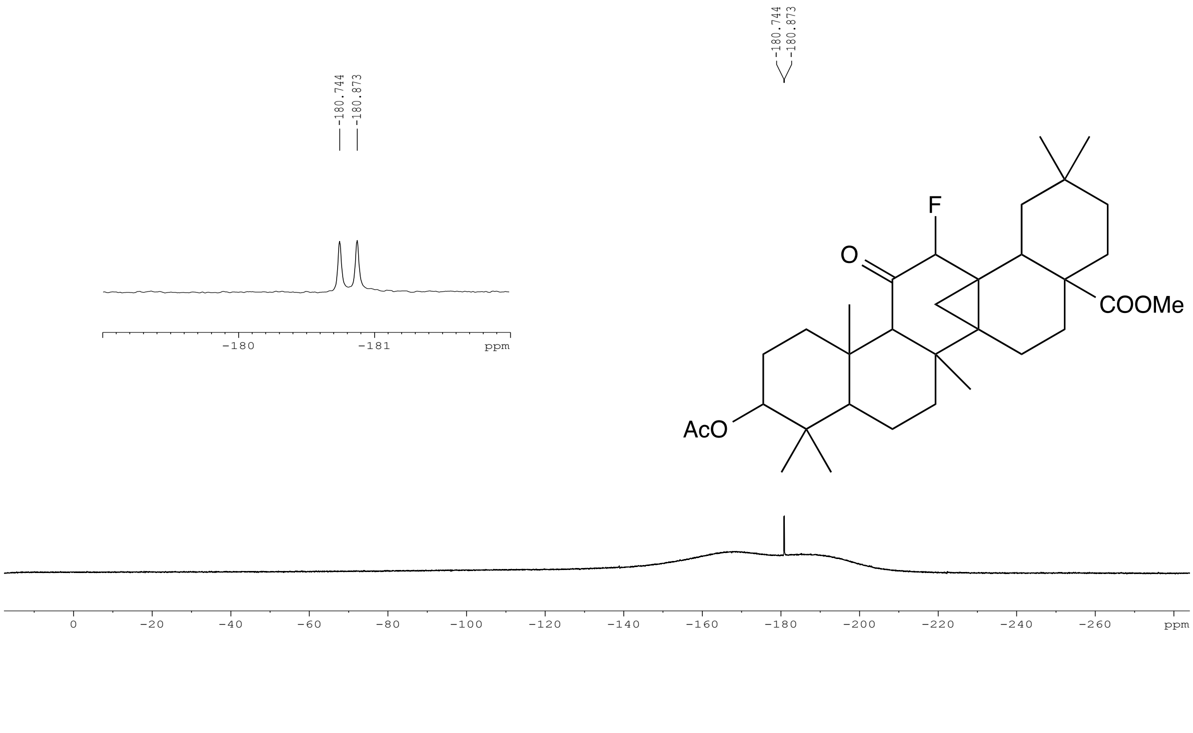

Compound 10: dehydrofluorination of 9 ^{1}H NMR (400 MHz, $CDCl_3$)

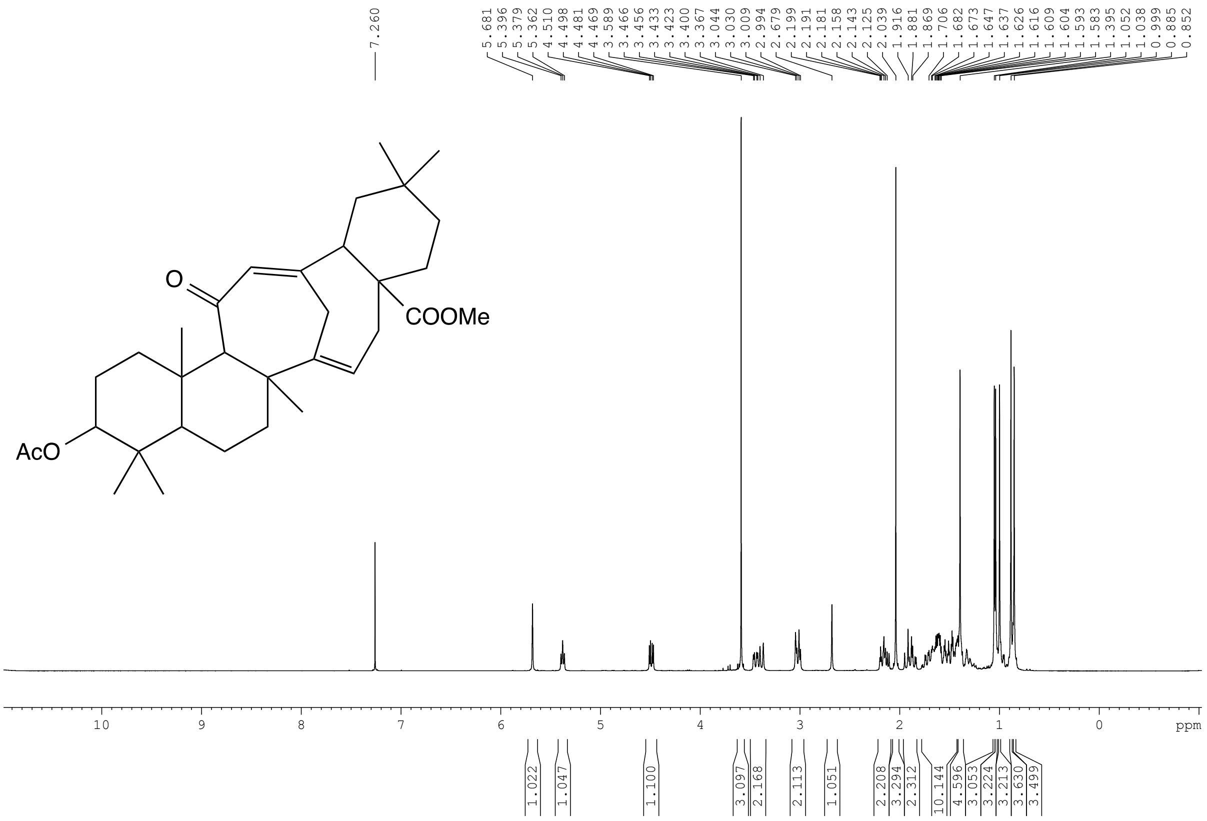

Compound 10: dehydrofluorination of 9 ^{13}C{1H} NMR (100 MHz, $CDCl_3$)

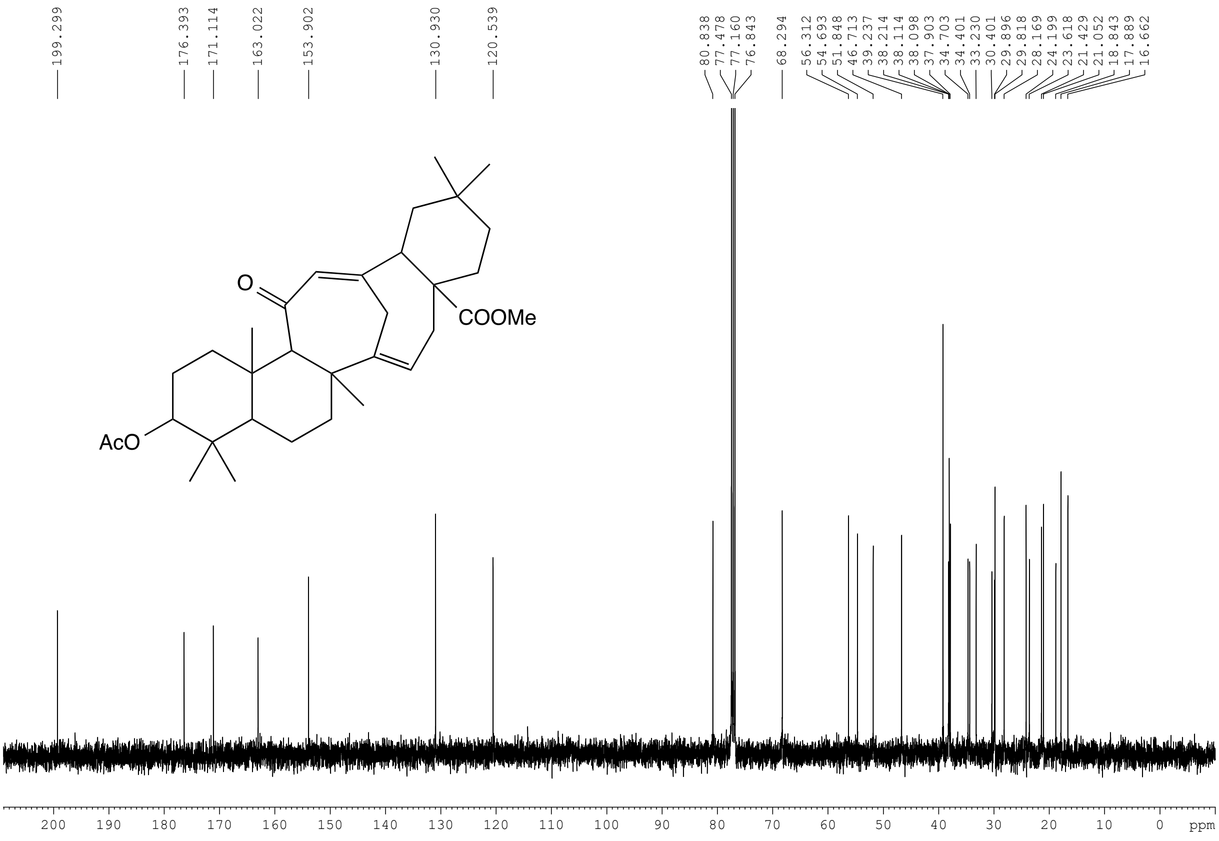

Compound 10: dehydrofluorination of 9 $^{13}C\{1H\}$ NMR(100 MHz, $CDCl_3$) (DEPT)

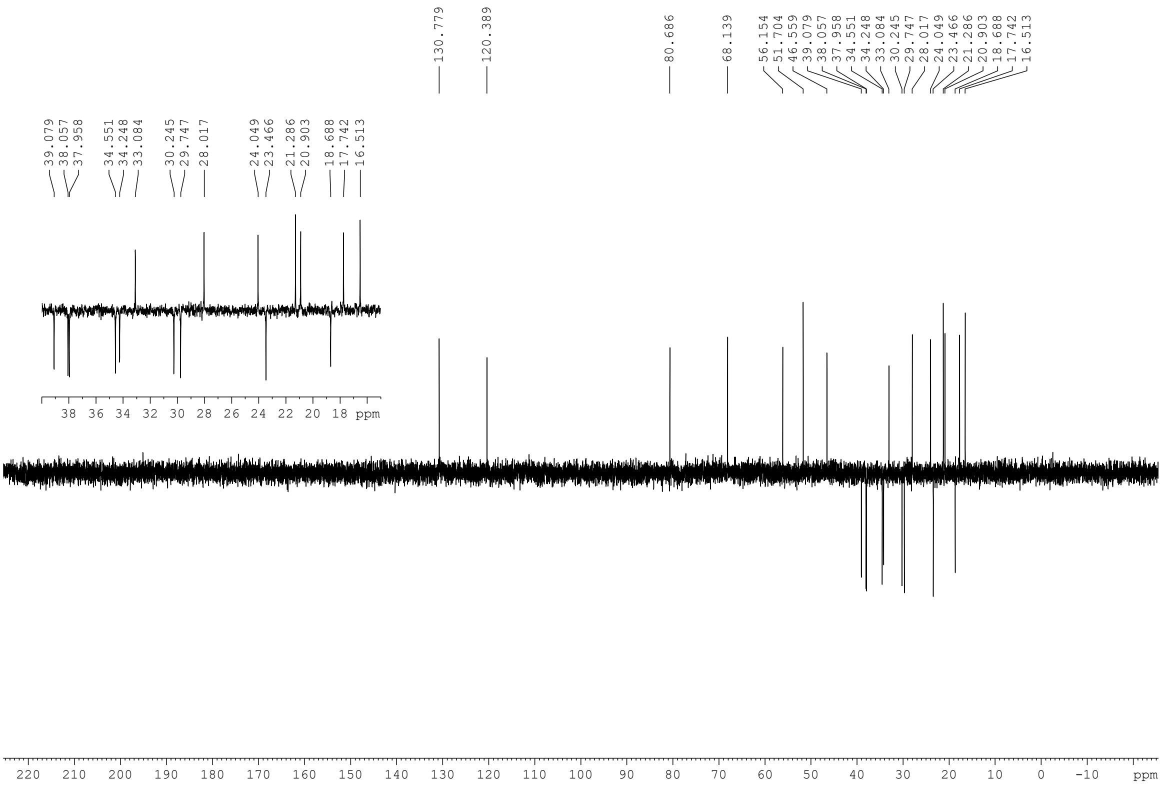

Section 2. Computational

Computational Methods

All calculations were performed with the development version of the Gaussian suite of programs (see refs 10-11 in the main paper). The wB97XD exchange-correlation density functional was used with the 6-31+g(d,p) basis set. NMR calculations were performed with a larger basis set 6-311++g(2df,2pd) on the 6-31+g(d,p) optimized geometries. Chemical shifts were obtained using as a reference value tetramethylsilane (TMS) optimized at the same level of theory with Td symmetry. Geometries were optimized in the presence of solvent simulated via the implicit SMD method,[1] using the default parameters for chloroform. To assure numerical consistency, a large integration grid was used with 99 shells and 590 angular points per shell (namely 'ultrafine') and the accuracy of the two-electron integrals was increased to 10^{-12} a.u. All the species were confirmed to be minima or transition states according to their number of imaginary frequencies. Intrinsic Reaction Coordinate (IRC) calculations were performed in the transition states to characterize the reaction profile. Unless otherwise stated, the energies in the text and figures correspond to Gibbs free energies (expressed in kcal/mol) calculated at a temperature of $25\ °C$ and a pressure of 1 atmosphere.

[1]. A. V. Marenich, C. J. Cramer, D.G. Truhlar, , *J. Phys. Chem. B,* **2009**, *113,* 6378-6396.

Computed NMR Spectra

To complement the measured NMR spectra we present in Figs. S1a and S1b the calculated ^{1}H NMR spectra of compounds **1** and **2**, respectively. The three peaks marked by red arrows in Fig. S1a (1.63, 1.47, and 0.82 ppm) correspond to the methyl 27 hydrogen atoms of compound **1**. Their average value of 1.31 ppm compares well with the experimental value of 1.22 ppm. Averaging of the computed values is required due to the free rotation of the C27 methyl group around the bond connecting it to the molecular backbone. The two peaks marked by red arrows in Fig. S1b (0.38 and 1.38 ppm) correspond to the two cyclopropyl hydrogen atoms of compound **2**. The lower value compares well with the experimental value of 0.47 ppm. Note that the other cyclopropyl hydrogen could not be identified in the experiment as it was somewhat de-shielded by the α-fluoro-carbonyl, thus appearing in a region where many other methylene hydrogens resonate. The good agreement between the calculated and measured NMR signals further validates our computational analysis.

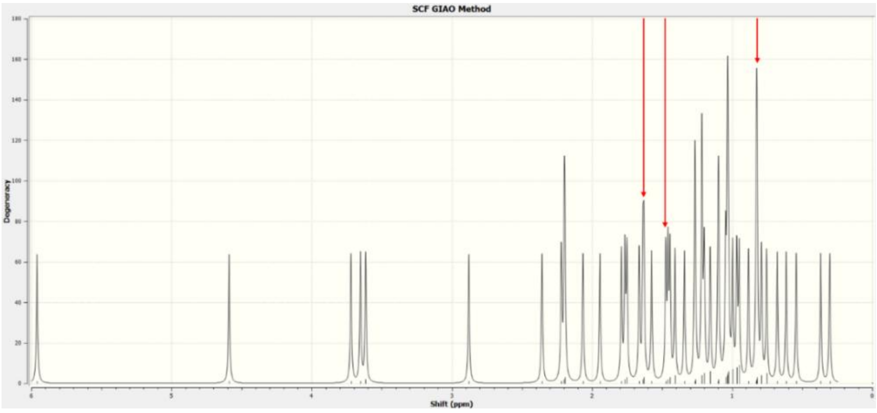

Figure S1a: Calculated ^{1}H NMR spectra of the Methyl-3-acetoxy-18β-Glycyrrhetate (**1**). The three red arrows mark the peaks associated with the C27 methyl group's hydrogen atoms. Computational details are given above.

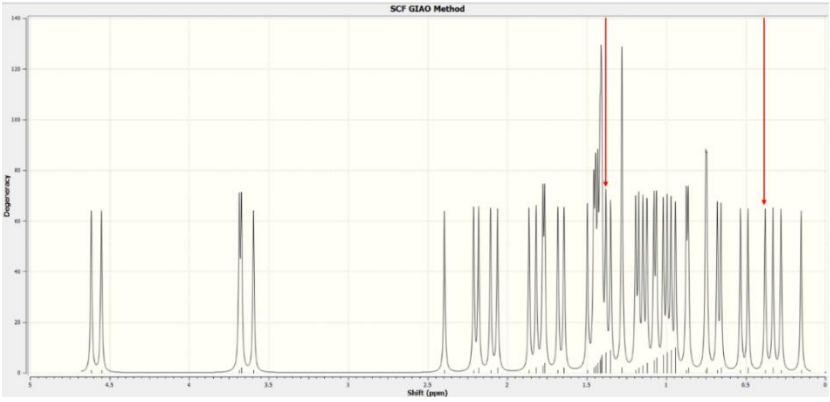

Figure S1b: Calculated ^{1}H NMR spectra of Methyl-3-acetoxy-12α–fluoro-13α,14α-cyclopropane-β-Glycyrrhetate (**2**). The two red arrows mark the peaks associated with the two cyclopropyl hydrogens in C27. Computational details are given above.

Analysis of the α and β-side attack

As discussed in the main text, the experimental findings and density functional theory (DFT) calculations agree that the α-side attack, where the C27 methyl group in **1** and the cyclopropane ring in **2** (see main text) are located**,** is the preferred reaction pathway (Fig S2). The optimized geometries of the pre-reaction molecular complexes are indeed nearly perpendicular to the triterpene's plane with F-F-C12 angle of 166.7^o and 179.6^o in the α or β sides, respectively (see Figure S2(a), (b)). Furthermore, the F-F distances after association are 1.401 and 1.398 Å respectively. These values are very close to the isolated F_2 molecule equilibrium distance of 1.392 Å and are considerably smaller than the C-F distances obtained for the α-side (2.841 Å) and β-side (2.192 Å) attacks. This indicates that the obtained geometries truly represent vdW molecular complexes between the triterpene and the F_2 molecules. Note that pre-reaction molecular complexes for both the α and the β faces (see below) present very similar energetic stability. The corresponding binding free-energies are 4.0 and 4.5 kcal/mol relative to the infinitely separated reactants (endoergic process) making the α-side approach slightly more energetically favorable.[1]

For completeness, we present here a computational analysis of the F_2 attack on the opposite side, denoted as the β side. The pre-reaction van der Waals (vdW) complex of **1** with the F_2 molecule located above the C12 position (Fig. S3a, b) is 4.5 kcal/mol higher in free-energy relative to the infinitely separated reactants. Similar to the α-side attack, the F_2 molecule attacks the double bond forming a transient α-fluorocarbocation that immediately and spontaneously reacts with the leaving fluoride anion (see Fig. S3c, d for the corresponding transition state). However, cyclopropanation is inhibited in the β-side attack since the C27 methyl group is spatially inaccessible. Instead, the leaving fluoride anion attacks the nearby C18 carbocation hydrogen resulting in the formation of the product **Pβ** with a double bond between carbons C13 and C18 (Fig. S3e, f). This species is less stable than the cyclopropanated geometry **2** by 1.4 kcal/mol (when both are coordinated to the leaving HF molecule) further supporting the preference of the α-side attack.

Having explored the pre-reaction complexation stage, we turn to examine the fluorination process. Using an implicit solvent model, we find that the reaction comprises two stages: (i) breaking the F_2 bond and attack of the adjacent fluorine atom on the C12 position; and (ii) the eventual abstraction of a hydrogen atom from the C27 methyl group by the remaining fluoride anion, resulting in the cyclopropane formation. For the first stage, we see that the energy-preferred transition state (TS) corresponding to the α-side bonding of the electrophilic fluorine atom to the C12 position has a free energy of 2.7 kcal/mol above the most stable F_2/**1** complex and 6.7 kcal/mol above the infinitely separated reactants (see SI section 4, Figure S2 c, d; Cartesian coordinates are available in the same section). The geometry and charge distribution of the favorable TS moiety, along with clear visual

evidence from the inspection of the imaginary frequency (see gif file Figure 3), all show the cleavage of the F_2 molecule (F-F distance of 1.485 Å) and the formation of a C-F bond on C12 with a bond length of 2.279 Å. This, in turn, leads to the formation of a leaving fluoride anion and an $\alpha-$fluorocarbocation in position C13, with a partial charge of +0.24e (full charge analysis can be found in Table S1 of SI section 2).

The first reaction stage ends once the F_2 bond is cleaved and the remaining fluoride anion is free to accept one of the C27 methyl hydrogen atoms, both located on the α-plane of the molecule. In the resulting ion-cage, the fluoride anion is close to the cation and the 27-methyl, causing the cyclopropanation reaction to occur without an energetic barrier in the absence of any explicit solvent molecule. Compound **2** and the leaving HF molecule are found to be 102 kcal/mol more stable than the separated reactants.

To explore the entire reaction path between the reactants and the cyclopropanated species (**2**) we performed intrinsic reaction coordinate (IRC) analysis.[2] We find that after 16 steps of the IRC procedure the C12-F bond is fully formed with bond length of 1.387 Å, compared to 1.372 Å in the most stable isolated α-fluorocarbocation geometry, and the fluoride anion is practically free (2.180 Å from the other fluorine) (see SI section 2, Figures S4 and S5). When comparing total electronic energy[3] this geometry, which lies in between TS and the products along the reaction coordinate, is found to be 39 kcal/mol more stable than TS and 73.0 kcal/mol less stable than the cyclopropanated product complex. Notably the exploration of alternative transition states corresponding to a mechanism lacking a cationic center, were unsuccessful. It should be further noted that the transition geometry is near that of the molecular complex with a very low barrier practically ruling out any competitive pathway.

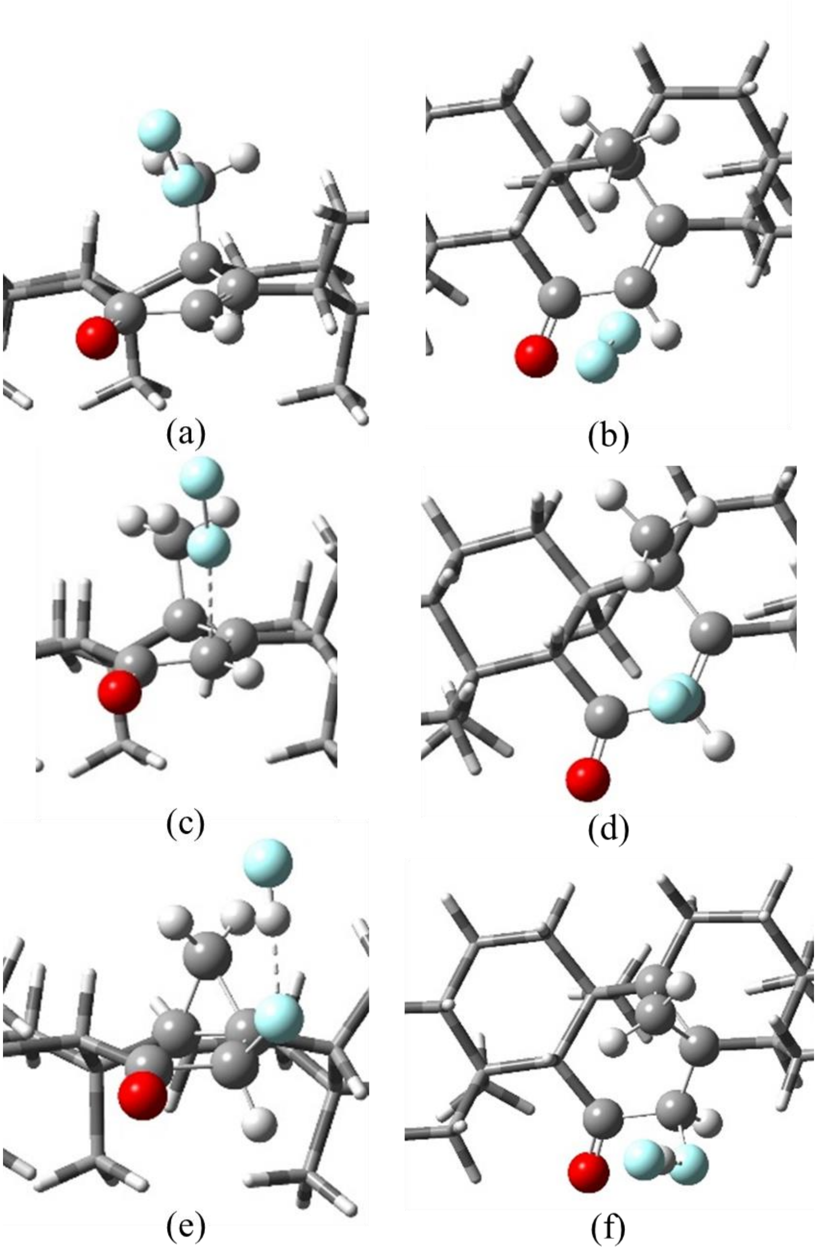

Figure S2. DFT optimized geometries of a few key structures along the α-side attack reaction path: side (left) and top (right) views are provided in the left and right columns respectively. (a) and (b) pre-reaction vdW complex; (c) and (d) fluorination transition state; (e) and (f) cyclopropane intermediate with the by-product HF molecule.

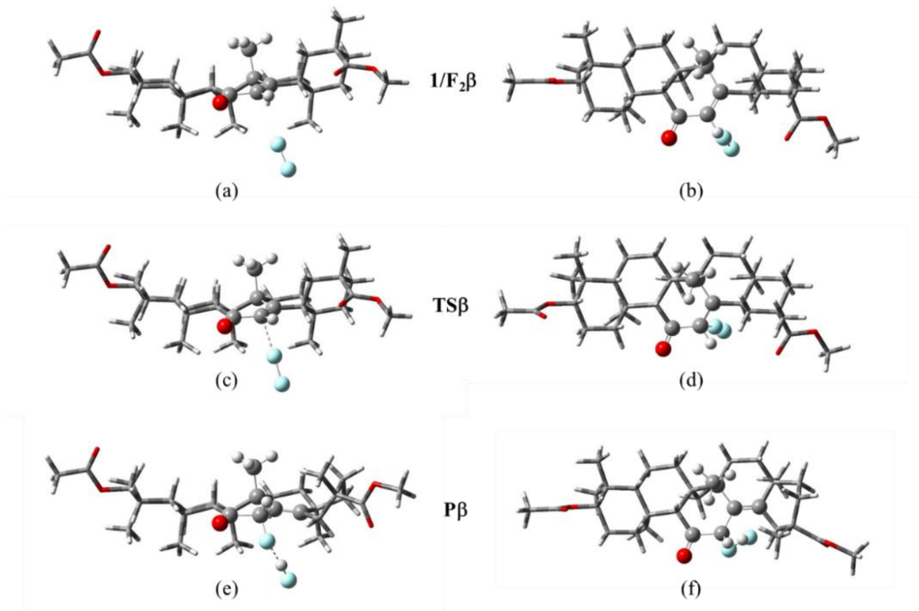

Figure S3: DFT optimized geometries of a few key structures on the reaction mechanism through the β-side attack: (a), (b) pre-reaction vdW complex **1/F₂β**; (c), (d) fluorination transition state **TSβ**; (e), (f) the product **Pβ** with the by-product HF molecule. Side and top views are provided in the left and right columns, respectively.

<u>Intrinsic reaction coordinate analysis</u>

In the main text the results of the intrinsic reaction coordinate calculation were provided. For completeness, we present in Fig. S4 the corresponding geometry (panels a and b) and potential energy profile (panel c).

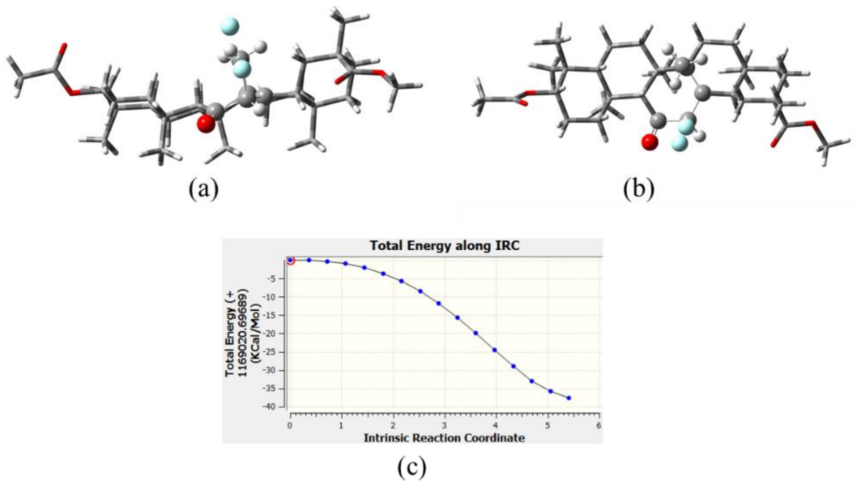

Fig S4. Side (a) and top (b) views of the DFT optimized geometry of the 16th step structure of the IRC calculation starting from the fluorination transition state **TSα** (see main text) toward the products. The corresponding potential energy profile is given in panel (c).

Isolated α-fluorocarbocation geometry

Following the F_2 attack, the fluoride anion has an important role in the formation of the cyclopropanated intermediate. Nevetheless, it is instructive to study the properties of the unstable carbocation in the absence of the leaving fluoride anion, thus preventing the fluoride attack and the cyclopropanation. For the corresponding α-side attack structure, we identified two different conformers depending on the two spatial conformations of ring C, with an exchange of the axial/equatorial character of the F and H substituents at C12 (see Fig. S5) leading also to variations in the atomic partial charges (see Table S1). Due to the vertical orientation of the F_2 attack, the axial isomer is expected to form first. However, the conformer with the axial F (**Icat^{ax}**) is found to be less stable by 6.5 kcal/mol in free energy than the conformer with equatorial fluorine (**Icat^{eq}**). Therefore, one would expect that once the C12-F bond forms the two conformers undergo a fast "chair-boat" interconversion. Importantly, this interconversion is accompanied by tilting of the C27 methyl group towards the C13 position, such that the F-C14-C13 angles varies from 105.4 to 87.7 degrees in a way that facilitates the cyclopropanation process. We therefore conclude that even in the absence of a base to cleave a proton from the C27 methyl group the cyclopropanation process is promoted (but cannot be completed).

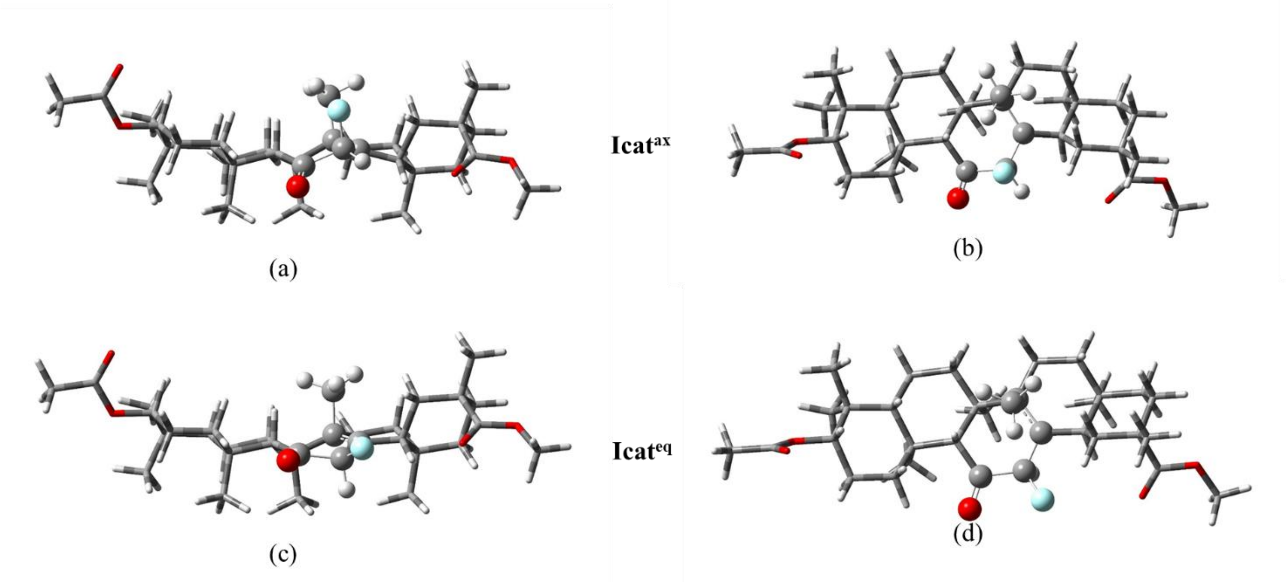

Figure S5: Side (left) and top (right) views of DFT optimized geometries of the axial (a), (b) and equatorial (c), (d) conformers of the isolated α-fluorocarbocation intermediate formed through the α-side attack.

Additionally, the isolated fluorinated cationic intermediate formed by the β-side attack has only one conformer with the coordinated fluoride positioned at the equatorial position. This structure was found to be 3.6 kcal/mol more stable than the **Icat^{eq}** isomer obtained in the α-side attack.

Nevertheless, this structure is irrelevant because an exchange from **Icat^{eq}** to this isomer would require a slow chiral inversion of the C12 that cannot compete with the spontaneously formation of the cyclopropanated species.

<u>Natural charge analysis</u>

As mentioned in the main text, partial charge analysis of the various compounds discussed in this study has been performed. The results of this analysis appear below.

Table S1a: Natural Population Analysis (NPA) partial charges on selected atoms of different geometries along the reaction path.

	**NPA Charges**						
	**1**	**1/F$_2$α**	**TSα**	**Icat^{ax}**	**Icat^{eq}**	**2/HF**	**2**
F1		0.002	-0.052	-0.353	-0.358	-0.414	-0.407
F2		-0.011	-0.114			-0.630	
O	-0.622	-0.620	-0.606	-0.534	-0.520	-0.568	-0.577
C CO	0.591	0.594	0.601	0.622	0.615	0.618	0.619
C12	-0.376	-0.391	-0.369	0.023	0.026	0.113	0.119
C13	0.158	0.173	0.235	0.478	0.589	-0.115	-0.109
C14	-0.111	-0.113	-0.120	-0.109	-0.180	-0.030	-0.033
C27	-0.698	-0.697	-0.694	-0.686	-0.683	-0.466	-0.466
C18	-0.308	-0.310	-0.318	-0.366	-0.386	-0.258	-0.256
H(Me-27)	0.254	0.253	0.258	0.302	0.273		
	0.251	0.250	0.251	0.292	0.270	0.264	0.259
	0.252	0.253	0.254	0.311	0.281	0.261	0.259
H (C13)	0.264	0.268	0.282	0.336	0.285	0.273	0.259
H(HF)						0.600	

Table S1b: Same as table S1a, where the charge variations with respect to the partial charges of reactant **1** (appearing in the second column) are presented to make it easier to follow the charge evolution along the reaction.

	**NPA Charges relative to 1**						
	**1**	**$1/F_2\alpha$**	**TSα**	**$Icat^{ax}$**	**$Icat^{eq}$**	**2/HF**	**2**
F1		0.002	-0.052	-0.353	-0.358	-0.414	-0.407
F2		-0.011	-0.114			-0.630	
O	0.000	0.003	0.016	0.089	0.102	0.054	0.045
C CO	0.000	0.003	0.009	0.030	0.024	0.027	0.028
C12	0.000	-0.015	0.007	0.399	0.402	0.489	0.495
C13	0.000	0.015	0.077	0.319	0.430	-0.273	-0.267
C14	0.000	-0.002	-0.009	0.002	-0.068	0.082	0.078
C27	0.000	0.001	0.004	0.012	0.015	0.233	0.232
C18	0.000	-0.002	-0.010	-0.058	-0.078	0.050	0.052
H(Me-27)	0.000	-0.001	0.004	0.048	0.019		
	0.000	0.000	0.001	0.041	0.019	0.013	0.009
	0.000	0.000	0.002	0.059	0.029	0.009	0.007
H (C13)	0.000	0.004	0.019	0.073	0.022	0.010	-0.005
H(HF)						0.346	

HF attack

In the main text, we compared results of the F_2 attack to those of the HF attack. For completeness we present here the corresponding molecular geometries (see Fig. S6) and their charge analysis (Tab. S2) for the isolated carbocations (without the leaving fluoride anion). The carbocation geometries obtained by α-side HF attack and F_2 attack are very similar. The main difference is the significant reduction of the movement of the C27 toward the cyclopropanated position when changing the stereoisomer from axial to equatorial (from 2.43 Å to 2.12 Å with F and from 2.38 Å to 2.31 Å with H). Consistently, both conformers are almost isoenergetic (the axial is 0.4 kcal/mol more stable).

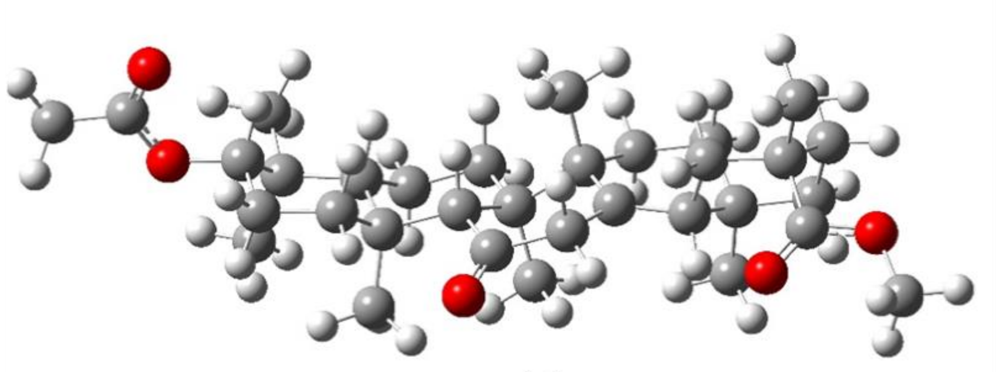

(a)

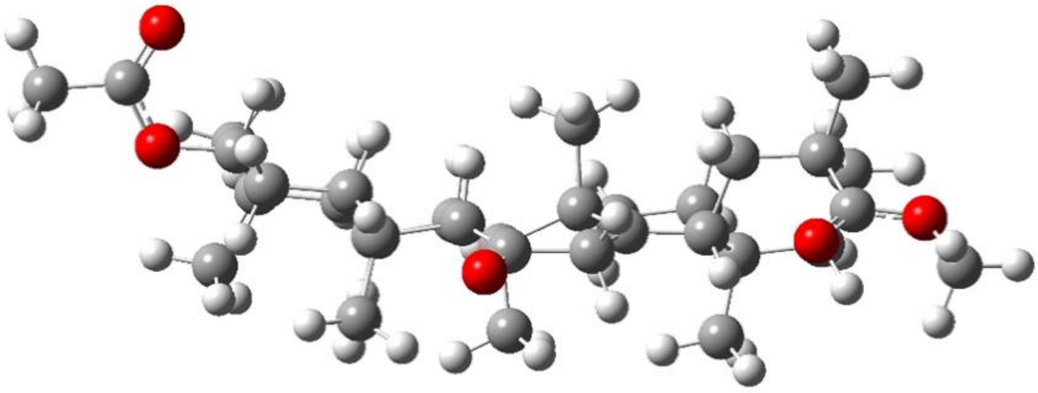

(b)

Figure S6: DFT optimized geometries of the (a) axial and (b) equatorial conformers of the isolated carbocation intermediate formed by the HF attack on **1**, depending on the orientation of the inserted H atom relative to the molecular plane.

Upon formation of the carbocation the global charge of the isolated molecule changes to +1 with respect to the neutral reactants. As a result, the charges of the atoms adjacent to the H or F addition site change significantly (see Tab. S2). For example, when fluorine is added to the double bond the alpha C12 charge changes negative (-0.376e) to positive (+0.023e / +0.026e depending on the isomer). In contrast, when H is added to the double bond the C12 charge becomes more negative (-0.707e / -0.719e depending on the isomer). Note, however, that this is a consequence of the C-F bond polarity and the overall charge of the CH_2 or CHF fragment remains almost null (between 0.006 and -0.046).

Notably, the C13 group, which is directly involved in the cyclopropanation reaction, is the only other carbon atom showing a significant charge variation (we only consider partial charge variations above 0.1 e) during the formation of the carbocation. The C13 partial charge of 0.158e in reactant **1** changes to 0.661e (for both isomers) with the H addition and to 0.478e and 0.589e with the F addition for the equatorial and axial isomers, respectively.

Table S2: Natural Population Analysis (NPA) partial charges on selected atoms, in both stereoisomers of the isolated carbocation obtained from the HF α-side attack. For comparison purposes, the results of the corresponding carbocation obtained from the F_2 attack are repeated here.

	**Absolute charges**					**Charges relative to 1**			
		**F**		**H**		**F**		**H**	
	**1**	**Icat^{ax}**	**Icat^{eq}**	**Icat^{ax}**	**Icat^{eq}**	**Icat^{ax}**	**Icat^{eq}**	**Icat^{ax}**	**Icat^{eq}**
F/H		-0.353	-0.358	0.372	0.358	-0.353	-0.358	0.372	0.358
O	-0.622	-0.534	-0.520	-0.554	-0.570	0.089	0.102	0.068	0.053
C CO	0.591	0.622	0.615	0.659	0.652	0.030	0.024	0.068	0.060
C12	-0.376	0.023	0.026	-0.707	-0.719	0.399	0.402	-0.331	-0.343
C13	0.158	0.478	0.589	0.661	0.661	0.319	0.430	0.503	0.503
C14	-0.111	-0.109	-0.180	-0.184	-0.176	0.002	-0.068	-0.072	-0.064
C27	-0.698	-0.686	-0.683	-0.684	-0.686	0.012	0.015	0.014	0.012
C18	-0.308	-0.366	-0.386	-0.393	-0.394	-0.058	-0.078	-0.085	-0.086
H(Me-27)	0.254	0.302	0.273	0.261	0.276	0.048	0.019	0.007	0.022
	0.251	0.292	0.270	0.272	0.277	0.041	0.019	0.021	0.027
	0.252	0.311	0.281	0.285	0.292	0.059	0.029	0.033	0.040
H (C13)	0.264	0.336	0.285	0.326	0.367	0.073	0.022	0.063	0.104

Section 3. Experimental X-Ray, CIF files

The X-Ray data for four compounds is deposited in “The Cambridge Crystallographic Data Centre (CCDC)” structures@ccdc.cam.ac.uk and can be viewed there.

1) For data on **Compound 2: Methyl-3-acetoxy-12α–fluoro-13α,14α-cyclopropane-β-Glycyrrhetate** (**2**) (roz2) CCDC - 2002977
2) For data on **Compound 3: (dehydrofluorination of 2)** (roz3) CCDC - 2002953
3) For data on **Compound 8: (dehydrofluorination of 7) (**roz5) CCDC - 2002979
4) For data on **Compound 10: (dehydrofluorination of 9)** (roz4) CCDC - 2002978

Section 4. Cartesian coordinates of the calculated structures

1/F₂α

E: -1862.95413134 H: -1862.118317 G: -1862.237808
C -2.784471 -2.408769 -0.353177
H -2.886485 -2.969782 0.581854
H -3.342789 -2.971780 -1.105986
C -3.352529 -0.995365 -0.208581
H -3.067400 -0.462987 -1.132101
C -1.324671 -2.352887 -0.796448
H -0.924144 -3.369805 -0.874556
H -1.313807 -1.936573 -1.808061
C -0.415432 -1.528696 0.138494
C -1.105052 -0.179377 0.531439
C -2.30620543 -0.211260 0.928770
C -4.914165 -0.938995 -0.212018
C -3.148212 1.245532 0.899496
H -2.802828 1.726957 -0.026513
H -2.718222 1.809344 1.728310
C -5.298199 0.552826 -0.185442
H -5.000921 1.002104 -1.138989
C -4.671790 1.339641 0.950427
H -5.060727 0.974794 1.907495
H -4.974839 2.388632 0.865644
C -2.847433 -0.806459 2.338679
H -1.988818 -0.621506 2.985381
H -3.708186 -0.344383 2.827774
H -3.032466 -1.882565 2.323515
H -1.079969 0.438875 -0.377200
C -0.165441 -2.385370 1.400876
H 0.345297 -3.319559 1.155882
H 0.429614 -1.871888 2.159503
H -1.111375 -2.663120 1.865950
C -5.575468 -1.717497 0.936605
H -5.425311 -1.254159 1.912786
H -6.654070 -1.788831 0.767390
H -5.180930 -2.737407 0.985071
C -5.441028 -1.521903 -1.537803
H -6.498803 -1.269634 -1.665383
H -4.891394 -1.122292 -2.398242
H -5.366897 -2.612797 -1.561690
C 0.950696 -1.176806 -0.597818
C -0.187584 0.569751 1.495426
C 1.259888 0.460271 1.260374

C 1.824302 -0.346304 0.341694
H 1.879726 1.061994 1.921965
C 3.337009 -0.340653 0.208713
H 3.734788 -0.042115 1.186102
C 1.658706 -2.471010 -1.079675
H 1.512956 -3.279769 -0.361662
H 1.172477 -2.798426 -2.005172
C 3.159160 -2.317559 -1.329783
H 3.578010 -3.300927 -1.577717
H 3.330178 -1.683572 -2.207104
C 3.782573 0.753870 -0.788570
H 3.413232 1.719293 -0.432114
H 3.336671 0.585556 -1.772864
C 5.418453 -1.641300 -0.400063
H 5.937510 -1.429768 0.545460
H 5.781050 -2.621239 -0.734918
C 5.302142 0.820138 -0.996506
C 5.805943 -0.570170 -1.417323
H 6.891212 -0.555650 -1.551315
H 5.369401 -0.795247 -2.397820
C 5.630085 1.861805 -2.082730
H 5.272178 2.856956 -1.798069
H 6.707788 1.917591 -2.264684
H 5.139562 1.579026 -3.019927
O -6.739675 0.647486 -0.086809
O -0.590588 1.259896 2.427023
C 0.733372 -0.298520 -1.864720
H 0.510689 0.743995 -1.630061
H 1.636793 -0.302577 -2.478159
H -0.070808 -0.689354 -2.491679
C 3.906518 -1.742098 -0.115672
C 3.719887 -2.654556 1.106075
H 2.677949 -2.707700 1.434729
H 4.055356 -3.674726 0.883740
H 4.307181 -2.282507 1.953128
C -7.369935 1.609833 -0.767424
C 5.984303 1.304087 0.283070
O 5.436085 1.916360 1.178547
O 7.294437 1.032872 0.296979
C 8.033240 1.505758 1.429348
H 9.062063 1.190736 1.258223
H 7.976172 2.594682 1.496806
H 7.648820 1.060665 2.350080
F 0.423074 4.113883 -0.670054
F 0.878166 2.893604 -0.154988

C -8.852733 1.556714 -0.535003
H -9.063564 1.676959 0.531486
H -9.345112 2.347107 -1.100858
H -9.241484 0.581746 -0.842634
O -6.807264 2.417163 -1.482215

2

E: -1863.13116797 H: -1862.295362 G: -1862.405923
C 2.855420 2.340354 -0.380875
H 2.966451 2.911656 0.547622
H 3.390071 2.903822 -1.149992
C 3.449147 0.937977 -0.235893
H 3.175065 0.402775 -1.160812
C 1.386751 2.266272 -0.787762
H 0.982291 3.279421 -0.884706
H 1.323807 1.801951 -1.781511
C 0.502496 1.459185 0.181685
C 1.215732 0.102010 0.497802
C 2.730027 0.135909 0.897928
C 5.010888 0.905555 -0.236993
C 3.269797 -1.316229 0.846468
H 2.924261 -1.790358 -0.082904
H 2.856669 -1.897691 1.671792
C 5.413643 -0.581857 -0.232321
H 5.124357 -1.020239 -1.193326
C 4.794792 -1.393506 0.890538
H 5.181745 -1.040872 1.852951
H 5.107241 -2.437953 0.788267
C 2.952719 0.710400 2.315860
H 2.099373 0.502835 2.963726
H 3.821466 0.251661 2.792874
H 3.122723 1.788914 2.317577
H 1.211594 -0.458032 -0.446765
C 0.270717 2.261690 1.476208
H -0.286083 3.180487 1.276645
H -0.293228 1.689949 2.222242
H 1.211440 2.557993 1.939378
C 5.660459 1.675345 0.924009
H 5.522790 1.191068 1.891851
H 6.736901 1.769731 0.752946
H 5.247383 2.686723 0.992376
C 5.530818 1.515212 -1.553334
H 6.590464 1.273611 -1.685483
H 4.984046 1.125742 -2.420113
H 5.447959 2.605748 -1.558300

C -0.860997 1.204311 -0.525155
C 0.320631 -0.725878 1.393385
C -1.195999 -0.731467 1.109562
C -1.755369 0.066893 -0.027016
H -1.685711 -0.451052 2.046984
C -3.296914 0.176719 0.021137
H -3.603693 -0.167684 1.016050
C -1.558599 2.460268 -1.053162
H -1.408175 3.285770 -0.352703
H -1.075864 2.761105 -1.989529
C -3.064529 2.304591 -1.291667
H -3.489885 3.299937 -1.469106
H -3.246671 1.728663 -2.206912
C -3.938250 -0.794247 -0.986742
H -3.626206 -1.813669 -0.740876
H -3.568437 -0.588665 -1.996345
C -5.320296 1.683151 -0.313191
H -5.804704 1.437708 0.642561
H -5.614643 2.714477 -0.544680
C -5.467624 -0.719877 -1.075476
C -5.859712 0.734776 -1.379897
H -6.946974 0.831018 -1.452088
H -5.450467 0.988126 -2.365653
C -5.969892 -1.659967 -2.186635
H -5.689259 -2.698829 -1.982726
H -7.057957 -1.606119 -2.289973
H -5.520592 -1.368942 -3.141823
O 6.855077 -0.659666 -0.129933
O 0.691173 -1.399953 2.332994
C -1.048302 -0.021521 -1.368053
H -1.237551 -2.909070 -0.509194
H -1.665940 0.086905 -2.252808
H -0.257423 -0.749183 -1.502852
C -3.790680 1.640335 -0.110035
C -3.489041 2.398282 1.191883
H -2.437062 2.337292 1.478054
H -3.747884 3.459703 1.093606
H -4.077996 1.984031 2.017935
C 7.499026 -1.606669 -0.820598
C -6.101397 -1.206237 0.226558
O -5.540554 -1.871312 1.075092
O -7.392031 -0.862474 0.322788
C -8.090647 -1.321908 1.485217
H -9.107295 -0.943580 1.383159
H -8.094660 -2.413754 1.524025

H -7.628206 -0.924737 2.391979
F -1.028935 -3.388715 -1.291668
F -1.541658 -2.116334 0.937379
C 8.980353 -1.538262 -0.583827
H 9.190134 -1.670674 0.481435
H 9.483470 -2.314934 -1.159129
H 9.357988 -0.554584 -0.877224
O 6.947236 -2.411898 -1.545636

TSα

E: -1862.95298619 H: -1862.118738 G: -1862.233563
C 2.810196 2.367716 -0.351860
H 2.910098 2.928746 0.583304
H 3.367990 2.931785 -1.104223
C 3.378853 0.954286 -0.208379
H 3.092504 0.421610 -1.131183
C 1.351292 2.309749 -0.797936
H 0.949557 3.325709 -0.880380
H 1.345307 1.888857 -1.807344
C 0.439274 1.485812 0.134456
C 1.132546 0.138436 0.530375
C 2.647324 0.173523 0.930450
C 4.940133 0.895410 -0.212623
C 3.171331 -1.284517 0.906653
H 2.824622 -1.769515 -0.016652
H 2.741547 -1.844577 1.738671
C 5.321381 -0.597288 -0.181896
H 5.022633 -1.048755 -1.133890
C 4.694876 -1.380642 0.956722
H 5.085709 -1.014533 1.912497
H 4.996044 -2.430251 0.873509
C 2.878342 0.773730 2.338023
H 2.021108 0.596572 2.988100
H 3.737416 0.308373 2.826748
H 3.070488 1.848493 2.318334
H 1.106531 -0.485196 -0.373976
C 0.183698 2.335361 1.400045
H -0.297786 3.285155 1.157087
H -0.440159 1.827620 2.140583
H 1.125383 2.582368 1.889737
C 5.604427 1.676407 0.932589
H 5.456076 1.215960 1.910400
H 6.682711 1.746033 0.760843

H 5.211215 2.696954 0.978986
C 5.465990 1.473548 -1.540827
H 6.523941 1.221784 -1.667837
H 4.916481 1.070113 -2.399515
H 5.390829 2.564316 -1.568703
C -0.927450 1.141796 -0.608701
C 0.218812 -0.600919 1.499961
C -1.230790 -0.534685 1.226687
C -1.800724 0.291950 0.309509
H -1.855464 -1.101808 1.912220
C -3.313577 0.299647 0.200268
H -3.696514 -0.011074 1.179359
C -1.639068 2.444107 -1.067018
H -1.487074 3.241521 -0.338377
H -1.156789 2.783074 -1.989845
C -3.140124 2.297466 -1.309804
H -3.557365 3.284978 -1.542601
H -3.319393 1.673834 -2.192714
C -3.787589 -0.780340 -0.804558
H -3.429879 -1.753857 -0.463660
H -3.350660 -0.607221 -1.791828
C -5.393541 1.627239 -0.363817
H -5.903678 1.405917 0.584288
H -5.749062 2.616464 -0.677828
C -5.310134 -0.822938 -0.993474
C -5.803043 0.576909 -1.393011
H -6.889716 0.575750 -1.515513
H -5.373736 0.810603 -2.374592
C -5.660925 -1.849888 -2.086499
H -5.312638 -2.852008 -1.814936
H -6.740882 -1.889944 -2.258061
H -5.175706 -1.564207 -3.025499
O 6.762512 -0.694124 -0.083350
O 0.607615 -1.246283 2.465182
C -0.711537 0.293514 -1.896905
H -0.396295 -0.729998 -1.694786
H -1.643298 0.237283 -2.462654
H 0.024821 0.761402 -2.552970
C -3.877629 1.710972 -0.096183
C -3.672860 2.602311 1.138292
H -2.627944 2.646932 1.457865
H -4.006042 3.627148 0.935529
H -4.253438 2.219331 1.984980
C 7.392350 -1.653341 -0.769197
C -5.983991 -1.308165 0.290210

O -5.427258 -1.904808 1.190866
O -7.297111 -1.052480 0.302598
C -8.029954 -1.524750 1.439229
H -9.062687 -1.224504 1.265210
H -7.958838 -2.612176 1.516300
H -7.651364 -1.066457 2.355903
F -0.839545 -3.661751 -0.813022
F -1.062909 -2.376127 -0.105020
C 8.875010 -1.602822 -0.535409
H 9.084956 -1.729633 0.530492
H 9.367055 -2.390330 -1.105553
H 9.264839 -0.626398 -0.837004
O 6.829397 -2.455917 -1.488880

Pβ

E: -1863.12800798 H: -1862.291437 G: -1862.405058
C 2.900416 2.198954 -0.152370
H 2.938307 2.585133 0.871814
H 3.490631 2.891199 -0.758292
C 3.490024 0.790339 -0.223587
H 3.293441 0.434794 -1.249515
C 1.469809 2.216656 -0.681845
H 1.065061 3.230480 -0.596081
H 1.507257 1.983494 -1.752450
C 0.521957 1.237517 0.036598
C 1.210731 -0.157912 0.175751
C 2.694776 -0.202904 0.687433
C 5.048620 0.757393 -0.109617
C 3.263146 -1.620649 0.422896
H 2.998616 -1.921300 -0.601044
H 2.797166 -2.341257 1.094956
C 5.472005 -0.705953 -0.337198
H 5.261928 -0.967258 -1.379891
C 4.780916 -1.702745 0.573351
H 5.085007 -1.525345 1.610773
H 5.115814 -2.712762 0.314583
C 2.789712 0.116128 2.195605
H 1.911445 -0.247727 2.732472
H 3.654498 -0.372678 2.648572
H 2.883869 1.184406 2.398758
H 1.272207 -0.567246 -0.841612
C 0.180017 1.818656 1.425698
H -0.550235 2.627898 1.347923
H -0.235663 1.078226 2.114492

H 1.066128 2.231018 1.910593
C 5.596860 1.309643 1.216125
H 5.393185 0.661910 2.070051
H 6.681692 1.433877 1.148227
H 5.165125 2.292337 1.429940
C 5.658521 1.591810 -1.252963
H 6.727627 1.375152 -1.346146
H 5.181563 1.365124 -2.213869
H 5.564805 2.665844 -1.069354
C -0.805824 1.051419 -0.807132
C 0.262135 -1.106055 0.887073
C -1.273216 -0.944907 0.728445
C -1.822568 0.264108 0.012728
H -1.644810 -1.885135 0.308532
C -3.142285 0.520057 0.035402
H -2.410972 0.410165 2.903821
C -1.377241 2.418244 -1.238438
H -1.275107 3.147054 -0.429979
H -0.782440 2.800472 -2.074745
C -2.842643 2.352636 -1.656085
H -3.189139 3.357433 -1.926795
H -2.951055 1.737032 -2.557194
C -4.187361 -0.414844 0.591382
H -4.858091 0.141561 1.251974
H -3.773906 -1.227969 1.187286
C -5.143039 1.460588 -1.147579
H -5.917788 1.567656 -0.378682
H -5.379893 2.190419 -1.930057
C -5.011908 -1.015620 -0.578163
C -5.179230 0.043270 -1.708634
H -6.101366 -0.141902 -2.268219
H -4.354895 -0.088917 -2.420521
C -4.309658 -2.260790 -1.136588
H -4.279849 -3.069569 -0.398923
H -4.807388 -2.632000 -2.036059
H -3.279947 -1.998762 -1.402994
O 6.902784 -0.799607 -0.141317
O 0.609039 -2.026864 1.597616
C -0.573946 0.225983 -2.103572
H -0.444416 -0.845501 -1.923844
H -1.446430 0.320686 -2.754572
H 0.297573 0.579214 -2.664098
C -3.749066 1.791837 -0.554169
C -3.921451 2.826367 0.574108
H -2.960113 3.136977 0.992936

H -4.433726 3.715145 0.186528
H -4.523513 2.422482 1.393879
C 7.610383 -1.605552 -0.939675
C -6.407186 -1.369832 -0.067929
O -6.923667 -0.928683 0.939764
O -7.044988 -2.210694 -0.890652
C -8.390046 -2.553560 -0.536709
H -8.731413 -3.235280 -1.315018
H -8.416054 -3.047374 0.437337
H -9.018938 -1.660410 -0.512415
F -2.765151 1.063054 3.477803
F -1.736245 -0.955711 2.087255
C 9.069481 -1.574395 -0.586278
H 9.205443 -1.911274 0.445464
H 9.628544 -2.220771 -1.262289
H 9.446061 -0.549772 -0.653481
O 7.125540 -2.271450 -1.834494

1/F2β

E: -1862.95390352 H: -1862.117944 G: -1862.237111
C -2.878772 -0.728841 -2.110686
H -2.937474 -1.821973 -2.076967
H -3.455247 -0.423036 -2.987976
C -3.466695 -0.109285 -0.841236
H -3.235483 0.968079 -0.901603
C -1.437711 -0.266870 -2.313126
H -1.023538 -0.737359 -3.211959
H -1.470614 0.808510 -2.512590
C -0.508831 -0.568914 -1.119232
C -1.202771 -0.182315 0.229310
C -2.700745 -0.596440 0.432414
C -5.028081 -0.154301 -0.783679
C -3.258119 0.204796 1.636588
H -2.965260 1.258489 1.523614
H -2.802152 -0.153318 2.560240
C -5.442489 0.622104 0.481008
H -5.201343 1.679589 0.330212
C -4.779316 0.139406 1.757048
H -5.115777 -0.878189 1.984640
H -5.106130 0.772749 2.588773
C -2.855317 -2.110283 0.710439
H -1.973113 -2.509743 1.212815
H -3.703486 -2.306339 1.370266
H -3.019472 -2.695739 -0.196533

H -1.225910 0.916801 0.242501
C -0.206233 -2.084128 -1.152635
H 0.328480 -2.364017 -2.063478
H 0.388308 -2.423526 -0.302787
H -1.131604 -2.659825 -1.148116
C -5.619923 -1.573053 -0.799208
H -5.440648 -2.125032 0.124677
H -6.702604 -1.524277 -0.949237
H -5.198224 -2.154882 -1.624886
C -5.603774 0.606177 -1.994216
H -6.670560 0.801106 -1.842910
H -5.100593 1.569463 -2.139104
H -5.511839 0.029967 -2.919224
C 0.828539 0.285215 -1.235787
C -0.260644 -0.526828 1.382512
C 1.180605 -0.361820 1.145480
C 1.725170 -0.029704 -0.039497
H 1.811382 -0.534650 2.015277
C 3.232172 0.131783 -0.123549
H 3.660040 -0.514853 0.651975
C 1.530135 0.020700 -2.594156
H 1.412307 -1.022918 -2.890772
H 1.018124 0.611807 -3.361363
C 3.021424 0.358924 -2.614468
H 3.439494 0.051992 -3.581387
H 3.161721 1.444059 -2.552270
C 3.632417 1.578018 0.248834
H 3.256664 1.797230 1.252529
H 3.162573 2.293652 -0.431506
C 5.303667 0.005807 -1.566181
H 5.848495 -0.662089 -0.884334
H 5.664326 -0.227970 -2.575741
C 5.144601 1.834378 0.185818
C 5.652945 1.450153 -1.213488
H 6.734277 1.600271 -1.278414
H 5.194438 2.142035 -1.930394
C 5.430161 3.321096 0.469858
H 5.064892 3.612841 1.460189
H 6.502235 3.535017 0.416781
H 4.921511 3.939718 -0.276808
O -6.878912 0.516046 0.628301
O -0.643167 -0.906261 2.485873
C 0.559797 1.818610 -1.169006
H 0.374591 2.175456 -0.153142
H 1.426120 2.365353 -1.547362

H -0.287872 2.106253 -1.794843
C 3.801846 -0.333007 -1.484626
C 3.660485 -1.858600 -1.597344
H 2.630625 -2.196527 -1.452084
H 3.993131 -2.205971 -2.583004
H 4.274945 -2.356108 -0.838985
C -7.569567 1.590570 1.022169
C 5.853266 1.045120 1.286563
O 5.325066 0.639150 2.303266
O 7.160632 0.892235 1.047316
C 7.920531 0.220088 2.058681
H 8.943651 0.193709 1.685302
H 7.874941 0.769997 3.001527
H 7.545256 -0.795136 2.207427
F 2.819719 -4.333473 1.542363
F 2.283125 -3.049114 1.411789
C -9.036084 1.284002 1.126169
H -9.194919 0.499900 1.872171
H -9.582979 2.181774 1.413022
H -9.407572 0.910632 0.167815
O -7.067347 2.671954 1.262247

TSβ

E: -1862.95129281 H: -1862.117049 G: -1862.229831
C 2.857099 1.988677 -1.029105
H 2.936217 2.763342 -0.259194
H 3.423112 2.358420 -1.888190
C 3.440258 0.664223 -0.533265
H 3.191740 -0.080365 -1.309040
C 1.406819 1.805926 -1.470112
H 0.998197 2.769069 -1.795246
H 1.417533 1.158970 -2.352393
C 0.491547 1.223409 -0.373072
C 1.183905 0.007305 0.328574
C 2.688575 0.156260 0.741568
C 5.002438 0.645468 -0.483915
C 3.240627 -1.255474 1.065810
H 2.929282 -1.947013 0.269852
H 2.799543 -1.621060 1.993334
C 5.410874 -0.788910 -0.098157
H 5.150253 -1.456449 -0.926377
C 4.763240 -1.300354 1.174281
H 5.116836 -0.711854 2.027916
H 5.084873 -2.332760 1.347560
C 2.862013 1.069431 1.977806

H 1.983992 1.028382 2.624646
H 3.711446 0.752487 2.587261
H 3.033530 2.115107 1.714616
H 1.192143 -0.797146 -0.421622
C 0.225560 2.359316 0.638099
H -0.351527 3.167912 0.183355
H -0.309317 2.032292 1.528227
H 1.164900 2.800089 0.970949
C 5.616236 1.686262 0.466430
H 5.448575 1.459362 1.520361
H 6.697521 1.742668 0.309527
H 5.201781 2.679383 0.266778
C 5.559161 0.913554 -1.895749
H 6.623442 0.659411 -1.933924
H 5.039918 0.313421 -2.652368
H 5.471267 1.967039 -2.175667
C -0.864527 0.700400 -1.019741
C 0.256769 -0.545171 1.409066
C -1.198158 -0.505429 1.143538
C -1.756818 0.101196 0.057331
H -1.808511 -1.107618 1.811098
C -3.256179 0.027706 -0.115887
H -3.684533 -0.044328 0.889879
C -1.566852 1.818127 -1.832062
H -1.454874 2.781394 -1.331906
H -1.050128 1.912789 -2.792896
C -3.055481 1.573614 -2.084370
H -3.478663 2.458190 -2.575797
H -3.187068 0.742319 -2.786007
C -3.634070 -1.288394 -0.840363
H -3.246436 -2.132616 -0.262819
H -3.168610 -1.331398 -1.827942
C -5.335643 1.082093 -1.083396
H -5.880600 1.097142 -0.129356
H -5.705451 1.938624 -1.660420
C -5.145321 -1.448203 -1.056864
C -5.668833 -0.216897 -1.814074
H -6.749331 -0.295371 -1.962729
H -5.211221 -0.225166 -2.810872
C -5.415956 -2.730688 -1.865971
H -5.042522 -3.616682 -1.341814
H -6.486694 -2.860782 -2.051116
H -4.907775 -2.666830 -2.833699
O 6.849645 -0.824116 0.056878
O 0.642940 -1.050524 2.455176

C -0.616657 -0.481491 -2.013662
H -0.467048 -1.441243 -1.513557
H -1.469966 -0.594208 -2.684707
H 0.254012 -0.284490 -2.641932
C -3.836957 1.293890 -0.790685
C -3.713033 2.485245 0.170899
H -2.683224 2.663107 0.492471
H -4.080695 3.401147 -0.306992
H -4.306011 2.311897 1.074922
C 7.517065 -1.900768 -0.370310
C -5.846558 -1.634847 0.288441
O -5.307309 -2.029115 1.303874
O -7.156298 -1.375131 0.223855
C -7.909411 -1.584668 1.424889
H -8.936330 -1.321217 1.174226
H -7.850822 -2.630141 1.736184
H -7.537575 -0.941559 2.225828
F -2.483312 1.943326 3.632014
F -1.981173 1.022070 2.535062
C 8.991299 -1.759681 -0.121448
H 9.174623 -1.649285 0.951265
H 9.516043 -2.637716 -0.497026
H 9.367609 -0.860199 -0.616884
O 6.989614 -2.863836 -0.893358

Icat^{ax}

E: -1763.07502956 H: -1762.239119 G: -1762.344599
C -2.719496 2.271148 -0.106616
H -2.743342 2.608005 -1.147763
H -3.293380 3.004920 0.464390
C -3.333671 0.876684 0.038700
H -3.126869 0.563463 1.075818
C -1.290416 2.290357 0.437062
H -0.857957 3.288288 0.314319
H -1.357349 2.098958 1.511287
C -0.360155 1.258998 -0.230296
C -1.086352 -0.126414 -0.316919
C -2.562600 -0.153059 -0.844255
C -4.891729 0.845356 -0.054507
C -3.120741 -1.565469 -0.541936
H -2.847090 -1.848473 0.483721
H -2.661903 -2.300086 -1.209487
C -5.311584 -0.613222 0.220341
H -5.081713 -0.844733 1.265746

C -4.640728 -1.642971 -0.672661
H -4.959427 -1.493563 -1.709686
H -4.978993 -2.641723 -0.378607
C -2.685572 0.120678 -2.361463
H -1.804861 -0.220085 -2.905644
H -3.534176 -0.423198 -2.779621
H -2.843733 1.175251 -2.593788
H -1.160761 -0.496339 0.713756
C 0.004835 1.742883 -1.647508
H 0.462654 2.732483 -1.633695
H 0.685890 1.061949 -2.172696
H -0.890238 1.826681 -2.260286
C -5.459484 1.362985 -1.385595
H -5.271208 0.692831 -2.225701
H -6.542649 1.489680 -1.302348
H -5.031368 2.339942 -1.631462
C -5.477883 1.712614 1.075905
H -6.550435 1.520582 1.179065
H -5.000458 1.493383 2.038283
H -5.362236 2.780768 0.870959
C 0.988386 1.143927 0.672514
C -0.143795 -1.087992 -1.000862
C 1.223896 -1.234201 -0.285920
C 1.845174 0.102454 0.076670
H 1.897662 -1.844268 -0.888910
C 3.274100 0.202218 -0.146317
H 3.534258 -0.353027 -1.060381
C 1.707979 2.511549 0.814727
H 1.515536 3.136382 -0.056914
H 1.265095 3.034790 1.665597
C 3.218166 2.400827 1.012442
H 3.643003 3.408982 1.052880
H 3.436314 1.930399 1.978471
C 3.813070 -0.772153 1.014581
H 3.423582 -1.779553 0.869331
H 3.477452 -0.408149 1.987137
C 5.424777 1.530764 0.099804
H 5.889974 1.183673 -0.832499
H 5.795566 2.548485 0.263023
C 5.347917 -0.806076 1.043536
C 5.879748 0.620955 1.234739
H 6.971596 0.607801 1.285372
H 5.517736 0.985124 2.203318
C 5.770360 -1.711700 2.214767
H 5.404075 -2.734549 2.081282

H 6.859833 -1.739355 2.297437
H 5.364911 -1.318931 3.151950
O -6.742749 -0.711064 0.052365
O -0.337695 -1.766056 -1.979275
C 0.686653 0.650865 2.140089
H 0.174670 -0.304829 2.205304
H 1.619480 0.576284 2.702381
H 0.079163 1.418010 2.619660
C 3.898029 1.609196 -0.115476
C 3.671681 2.272123 -1.482513
H 2.619479 2.295215 -1.770339
H 4.044911 3.301557 -1.469685
H 4.210717 1.726225 -2.263996
C -7.439348 -1.470127 0.908033
C 5.883324 -1.428322 -0.245770
O 5.194869 -1.939154 -1.110221
O 7.209439 -1.369549 -0.311432
C 7.826244 -1.950356 -1.471356
H 8.896400 -1.804975 -1.332666
H 7.590351 -3.014636 -1.533558
H 7.486600 -1.440720 -2.375600
F 0.943740 -1.942659 0.885240
C -8.902189 -1.463196 0.573215
H -9.459031 -2.005580 1.336728
H -9.267291 -0.435337 0.499712
H -9.053550 -1.940616 -0.399709
O -6.937881 -2.084355 1.829567

Icat^{eq}

E: -1763.08243983 H: -1762.247496 G: -1762.354929
C -2.783257 2.251899 0.023197
H -2.898156 2.658563 -0.986791
H -3.314118 2.937211 0.687515
C -3.381751 0.848924 0.117498
H -3.122501 0.479707 1.124768
C -1.315354 2.253750 0.439026
H -0.910391 3.265345 0.340733
H -1.265854 1.996887 1.504851
C -0.444896 1.285672 -0.385955
C -1.145537 -0.114912 -0.451935
C -2.664481 -0.148592 -0.852922
C -4.944538 0.835459 0.105969
C -3.224285 -1.561437 -0.545169
H -2.890978 -1.864396 0.457869

H -2.818380 -2.289127 -1.246749
C -5.367454 -0.623124 0.364316
H -5.088456 -0.885452 1.390324
C -4.749275 -1.625741 -0.590420
H -5.121346 -1.445856 -1.604881
H -5.073719 -2.633048 -0.309795
C -2.855765 0.163350 -2.353524
H -1.997592 -0.173315 -2.941337
H -3.728304 -0.354662 -2.755116
H -2.997057 1.227167 -2.553564
H -1.147970 -0.508152 0.575437
C -0.235718 1.906219 -1.785129
H 0.389832 2.799299 -1.724696
H 0.221560 1.230602 -2.511972
H -1.187627 2.218823 -2.210657
C -5.571768 1.390576 -1.182702
H -5.436752 0.735329 -2.044486
H -6.647331 1.529546 -1.041295
H -5.143396 2.366788 -1.430445
C -5.466615 1.678377 1.285378
H -6.529414 1.474123 1.448301
H -4.931445 1.445279 2.213566
H -5.373526 2.751233 1.094735
C 0.959710 1.145140 0.319233
C -0.261675 -1.122766 -1.159497
C 1.261926 -0.847879 -1.176701
C 1.843225 0.154652 -0.248847
H 1.454641 -0.368281 -2.155868
C 3.327373 0.144380 -0.082529
H 3.705351 -0.351970 -0.984810
C 1.605747 2.479689 0.733199
H 1.438361 3.204399 -0.066057
H 1.079316 2.859335 1.612105
C 3.103838 2.387488 1.027824
H 3.504351 3.404789 1.086152
H 3.269680 1.936336 2.012197
C 3.807801 -0.764629 1.087610
H 3.450586 -1.782937 0.918344
H 3.389490 -0.413977 2.032385
C 5.383863 1.577610 0.257541
H 5.909212 1.224869 -0.640348
H 5.716461 2.609346 0.418112
C 5.332927 -0.749241 1.260462
C 5.795599 0.706870 1.441508
H 6.881020 0.741052 1.565006

H 5.357681 1.082335 2.373986
C 5.695690 -1.587856 2.498842
H 5.384123 -2.630515 2.377538
H 6.773479 -1.566293 2.679778
H 5.192306 -1.180231 3.381415
O -6.806209 -0.704254 0.265940
O -0.617050 -2.135108 -1.711037
C 0.878818 0.213487 1.651239
H 0.986876 -0.870563 1.527433
H 1.614062 0.561571 2.370201
H -0.128737 0.384918 2.033022
C 3.872330 1.599513 -0.041252
C 3.675757 2.233371 -1.427016
H 2.630822 2.231401 -1.755736
H 4.017628 3.273731 -1.422155
H 4.255271 1.690133 -2.180846
C -7.464431 -1.490196 1.127537
C 6.019103 -1.392069 0.055175
O 5.449351 -1.990214 -0.836958
O 7.343760 -1.249575 0.101522
C 8.093884 -1.850651 -0.963811
H 9.138226 -1.639690 -0.738096
H 7.920223 -2.928442 -0.990963
H 7.815349 -1.407146 -1.922446
F 1.949438 -2.034932 -1.153690
C -8.941079 -1.472887 0.860610
H -9.467413 -1.995152 1.659065
H -9.301481 -0.444318 0.778987
H -9.138840 -1.972507 -0.092990
O -6.922143 -2.136643 2.002903

2

E: -1762.69071600 H: -1761.869049 G: -1761.975917
F 1.447446 -2.354144 -0.443445
O 5.564344 -2.158249 -0.739178
O -0.642918 -1.709585 -2.162743
O -6.912565 -2.254325 1.892188
C -8.942975 -1.506074 0.824718
H -9.451064 -2.179192 1.514693
H -9.160693 -1.798616 -0.206475
C -7.461033 -1.554629 1.062455
H 3.643303 -1.828182 1.042744
H 8.114994 -2.752284 -1.122362
H 9.125846 -1.276094 -1.195357

C -4.756343 -1.573291 -0.690918
H -5.143216 -1.334141 -1.687587
H -5.072152 -2.597718 -0.467247
C -3.230963 -1.494895 -0.656673
H -2.817816 -2.167576 -1.410219
H -2.886611 -1.860185 0.321137
C 5.994712 -1.500506 2.454344
H 5.550155 -1.079959 3.362422
H 7.083452 -1.441456 2.548348
H 5.706393 -2.555351 2.392396
O -6.812939 -0.721584 0.241471
O 7.416064 -1.048279 -0.147017
H -9.307793 -0.484425 0.964061
C -5.371176 -0.634360 0.330794
H -5.080403 -0.958451 1.335700
C -4.966015 0.843016 0.162381
C -3.404247 0.872114 0.156771
H -3.130236 0.452833 1.139987
C -2.687822 -0.060082 -0.873824
C -1.175527 -0.055584 -0.468695
H -1.173794 -0.494273 0.537554
C -0.278364 -0.981911 -1.259829
C 1.221978 -1.033745 -0.904767
H 1.758084 -0.958615 -1.856390
C 1.787265 -0.050695 0.083412
C 3.329760 0.043280 0.020704
H 3.632673 -0.431338 -0.920308
C 3.966667 -0.788552 1.149114
H 3.599919 -0.442985 2.121258
C 5.496835 -0.714761 1.227189
C 6.126515 -1.377959 0.003564
C 8.109256 -1.665807 -1.237226
H 7.640978 -1.400839 -2.188211
C 5.899575 0.764711 1.334454
H 6.987541 0.862576 1.393015
H 5.492988 1.150117 2.277809
C 5.364875 1.566300 0.151464
H 5.845556 1.191860 -0.763364
H 5.667387 2.617073 0.243099
C 3.834742 1.507806 -0.043376
C -0.455093 1.326544 -0.320080
C 0.904130 1.152140 0.416681
C 1.609799 2.461451 0.781337
H 1.129165 2.881946 1.671848
H 1.465562 3.194713 -0.016410

C 3.114451 2.326968 1.040442
H 3.290023 1.872159 2.022978
H 3.548278 3.333376 1.088775
C 1.081305 0.051346 1.422324
H 0.287871 -0.651408 1.648947
H 1.693408 0.282752 2.287682
C -1.337075 2.253996 0.537791
H -1.273872 1.920449 1.582830
H -0.929827 3.270226 0.504168
C -2.807121 2.280560 0.128753
H -2.920279 2.733750 -0.862488
H -3.339070 2.935006 0.824436
C -2.911135 0.349524 -2.347871
H -3.784517 -0.154745 -2.766869
H -2.061461 0.063833 -2.969213
H -3.074927 1.421922 -2.471176
C -5.616522 1.474227 -1.079227
H -6.692214 1.591326 -0.917584
H -5.483128 0.880158 -1.984520
H -5.200692 2.469414 -1.265765
C -5.482791 1.602874 1.399245
H -4.940353 1.309945 2.305858
H -6.544891 1.388062 1.555688
H -5.389305 2.686050 1.279949
C -0.211961 1.955017 -1.705296
H 0.355580 2.885100 -1.624474
H -1.147853 2.198622 -2.207430
H 0.348334 1.283079 -2.365830
C 3.537958 2.090236 -1.433728
H 4.125368 1.568504 -2.197661
H 3.802276 3.154166 -1.474938
H 2.486170 1.996725 -1.710831

[1]. Despite the formation of the reactant complex being slight endoergic the enthalpy of the association process was found to be slightly favorable (-1.3 and 1.0 kcal/mol for the α-side and β-side attacks, respectively). The unfavorable binding free energy must be considered in light of the fact that we performed these calculations in an implicit solvent model, such that the entropic contribution (mainly translational and rotational) is overestimated with respect to the real solvent environment relevant to the experiment. Therefore, due the change in the number of molecules when calculating the free-energy difference of the complexation process, we overestimate the entropy of the reactants over that of the product complex. The associated entropic penalty was previously estimated to be of the order of 15-20 kcal/mol, which is larger than the absolute free-energy values that we obtained. See also: Besora, M.; Vidossich, P.; Lledós, A.; Ujaque, G.; Maseras, F. Calculation of reaction free energies in solution: a comparison of current approaches *J. Phys. Chem. A* 2018, *122*, 1392-1399.

[2]. a) Fukui, K. The path of chemical-reactions – The IRC approach, *Acc. Chem. Res*., **1981**, *14,* 363-368; b) Hratchian, H. P.; Schlegel, H. B. Transition States, and Following Reaction Pathways on Ab Initio Potential Energy Surfaces. In *Theory and Applications of Computational Chemistry: The First 40 Years* (Eds.: Dykstra, C. E.; Frenking, G.; K. S. Kim, K. S.; Scuseria, G.), Elsevier, Amsterdam, **2005**, pp 195-249.

[3]. We use total electronic energy here and not free energy analysis including vibrational corrections, since the discussed geometry is a snapshot of the reaction pathway obtained by IRC and not a fully optimized geometry.
